# Integrative analyses of TEDDY Omics data reveal lipid metabolism abnormalities, increased intracellular ROS and heightened inflammation prior to autoimmunity for type 1 diabetes

**DOI:** 10.1186/s13059-021-02262-w

**Published:** 2021-01-21

**Authors:** Leandro Balzano-Nogueira, Ricardo Ramirez, Tatyana Zamkovaya, Jordan Dailey, Alexandria N. Ardissone, Srikar Chamala, Joan Serrano-Quílez, Teresa Rubio, Michael J. Haller, Patrick Concannon, Mark A. Atkinson, Desmond A. Schatz, Eric W. Triplett, Ana Conesa

**Affiliations:** 1 Microbiology and Cell Science Department, Institute for Food and Agricultural Sciences, University of Florida, Gainesville, USA; 2 Department of Pathology, Immunology and Laboratory Medicine, University of Florida Diabetes Institute, Gainesville, FL USA; 3 Gene Expression and RNA Metabolism Laboratory, Instituto de Biomedicina de Valencia (CSIC), Jaume Roig, 11, 46010 Valencia, Spain; 4 Laboratory of Neurobiology, Prince Felipe Research Center, Valencia, Spain; 5 Department of Pediatrics, University of Florida Diabetes Institute, Gainesville, FL USA; 6 University of Florida Genetics Institute, Gainesville, FL USA

## Abstract

**Background:**

The Environmental Determinants of Diabetes in the Young (TEDDY) is a prospective birth cohort designed to study type 1 diabetes (T1D) by following children with high genetic risk. An integrative multi-omics approach was used to evaluate islet autoimmunity etiology, identify disease biomarkers, and understand progression over time.

**Results:**

We identify a multi-omics signature that was predictive of islet autoimmunity (IA) as early as 1 year before seroconversion. At this time, abnormalities in lipid metabolism, decreased capacity for nutrient absorption, and intracellular ROS accumulation are detected in children progressing towards IA. Additionally, extracellular matrix remodeling, inflammation, cytotoxicity, angiogenesis, and increased activity of antigen-presenting cells are observed, which may contribute to beta cell destruction. Our results indicate that altered molecular homeostasis is present in IA-developing children months before the actual detection of islet autoantibodies, which opens an interesting window of opportunity for therapeutic intervention.

**Conclusions:**

The approach employed herein for assessment of the TEDDY cohort showcases the utilization of multi-omics data for the modeling of complex, multifactorial diseases, like T1D.

**Supplementary Information:**

The online version contains supplementary material available at 10.1186/s13059-021-02262-w.

## Introduction

Type 1 diabetes (T1D) manifests as a result of inappropriate activation of inflammation and the immune response against self-antigens, resulting in autoimmune destruction of pancreatic islet β cells. The onset of T1D is preceded by the appearance of islet autoantibodies against insulin (IAA), glutamic acid decarboxylase (GADA), zinc transporter 8 (ZnT8A), and/or insulinoma associated antigen-2 (IA2A). The Human Leukocyte Antigen (HLA) complex is the primary genetic contributor of T1D susceptibility, with the highest risk conferred by specific allelotypes of the class II genes DQA1, DQB1, and DRB1. The global incidence rate of T1D is increasing annually, with many new cases appearing in western, developed countries [1]. Progression of the disease is not well understood, because the death of pancreatic islet β cells, the primary pathology of T1D, is inherently difficult to measure. Furthermore, the interplay between genetic risk, physiological biomarkers, and environmental triggers is unclear. Multiple triggers are thought to influence disease progression rates, degradation of islet β cells, and ultimately, insulin dependence [2].

High-throughput omics approaches in human and rodent models, including genome-wide association, transcriptomics, metabolomics, proteomics, and microbiome analyses, have led to the discovery of features that are strongly associated with islet autoimmunity [3–8]. Comparison of T1D-specific transcriptional signatures in blood mononuclear cells to recent-onset patient cells facilitated detection of overexpressed interleukin-1 family members, which are involved in immunocyte chemotaxis and immune receptor activity [9]. Accordingly, metabolomics studies suggest that a stress signature that impacts levels of phospholipids, methionine, glutamate, and energy metabolites [10] is present before seroconversion (SC). However, the magnitude and direction of these metabolite changes are sometimes contradictory, possibly due to confounding factors like age, disease severity, or demographics. While many studies have discovered key biological processes that play an important role in T1D onset, these processes are rarely integrated to analyze their collective role in this multifactorial disorder.

The Environmental Determinants of Diabetes in the Young (TEDDY) study is a prospective study that was designed to identify T1D-associated environmental factors in children carrying high genetic risk for the disease [11]. TEDDY investigators measured a wide variety of environmental, demographic, and molecular factors in at-risk children from birth to T1D diagnosis. An integrative approach was applied to these datasets to model the relationship between disease state of subjects and their biomolecular profiles across time. Ultimately, the novel, multi-omics approach employed to assess the TEDDY cohort provides new insights regarding molecular signatures that characterize T1D, while informing a hypothetical model for disease progression. This model, which provides foundations for future studies, proposes that lipid metabolism impairment (LMI), glycolysis dysregulation, and accumulation of intracellular reactive oxygen species (ROS) may precede and exacerbate the autoimmune responses associated with T1D progression. More importantly, our study reveals that this molecular signature can be detected as early as 12 months before confirmed β cell autoimmunity, which opens an exciting opportunity for early diagnosis and therapeutic intervention.

## Results

### Overview of the data analysis approach

**Fig. 1:**
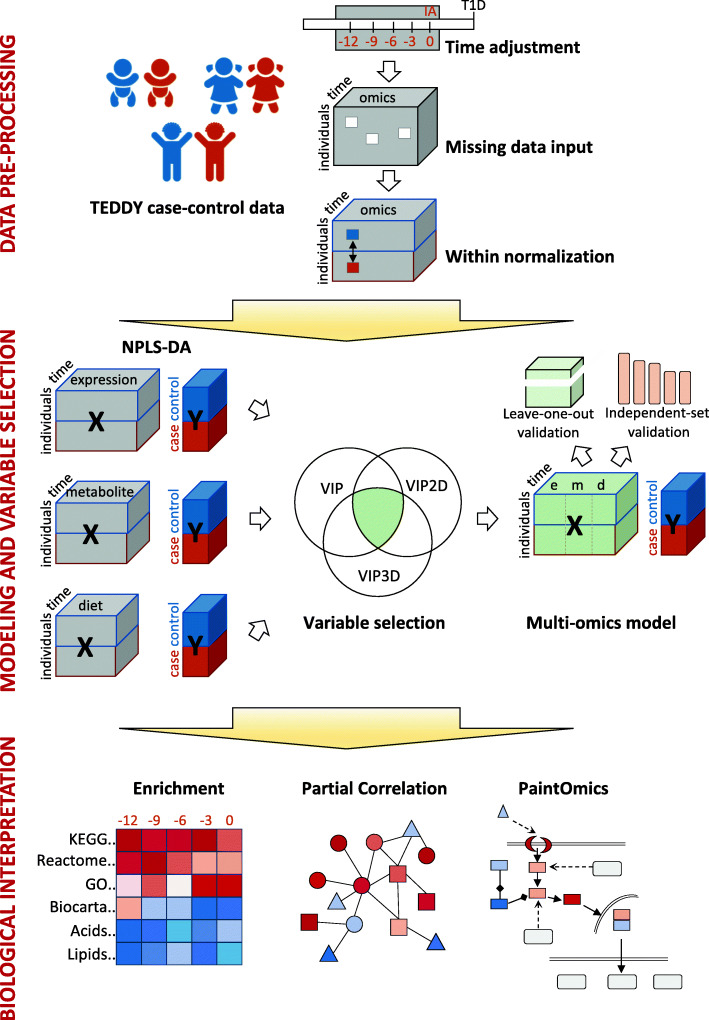
Overview of the data analysis strategy. The TEDDY case-control dataset was used. Preprocessing entailed data binning, missing value imputation, and within case-control normalization. Statistical analysis proceeded through NPLS-DA models on islet autoimmunity for each omics modality, selection of variables using the VIP statistics, and a final multi-omics NPLS-DA model with selected variables. Integrative biological interpretation used enrichment analysis to identify relevant pathways, partial correlation analysis to find novel gene/metabolite relationships, and PaintOmics3 to add pathway models to multi-omics data visualization

Metabolomics, transcriptomics, and dietary biomarker data were binned in 3-month intervals prior to seroconversion and arranged into a 3-way tensor structure where the dimensions were subjects (first mode), omics features (second mode), and time (third mode), with element *x*_ijk_ representing the value of the *j* omic variable in subject *i* at time point *k* (Fig. 1). Only subjects with complete case-control paired data for all omics measurements in at least 3 out of 5 time points were considered. A Tucker3 model-based approach was used to impute missing values within each dataset (Additional file 13: Fig. S1). After within-normalization, each omics tensor was analyzed by a multiblock strategy, N-way partial least squares-discriminant analysis (NPLS-DA), using the case (confirmed autoimmunity) and control labels as response variables. Features were selected by variable importance for projection (VIP) analyses. Selected genes, metabolites, and dietary biomarkers were joined to create an integrated NPLS-DA model to assess classification power and analyze the global dynamics of the multi-omics changes. Next, selected biomarkers were analyzed by partial correlation to identify associations among them and to create integrated metabolite/gene expression networks of disease progression. To aid in the interpretation of the results, enriched pathways/metabolite classes were identified, and data were visualized with PaintOmics3 [12, 13], which was manually edited to include associations found by partial correlation analysis.

### Multiblock analysis of multi-omics TEDDY data predicts the onset of autoimmunity

Our analysis strategy requires that multi-omics data are available for both the matched case-control pair at each binned time point. These resulted in the selection of 136 paired individuals (68 cases and 68 controls) that had complete measurements for at least 3 out of 5 time points and in the overall integration 476 blood gene expression, 680 citrate plasma metabolomics, and 680 plasma dietary biomarker datasets. A total of 170 individuals, with multi-omics data in less than 3 time points, were used for model validation at individual time points (Additional files 1, 2, 3, 4, 5, 6, 7, 8, 9, 10). Our final dataset consisted of 48% females and 52% males; 29% Swedish, 7% German, 33% North Americans, and 31% Finnish. The distribution of first-appearing autoantibody was 48% IAA, 31% GADA, 17% GADA_IAA, and 4% others (Additional file 11). This subject selection faithfully resembles the composition of the TEDDY cohort [14].

**Fig. 2:**
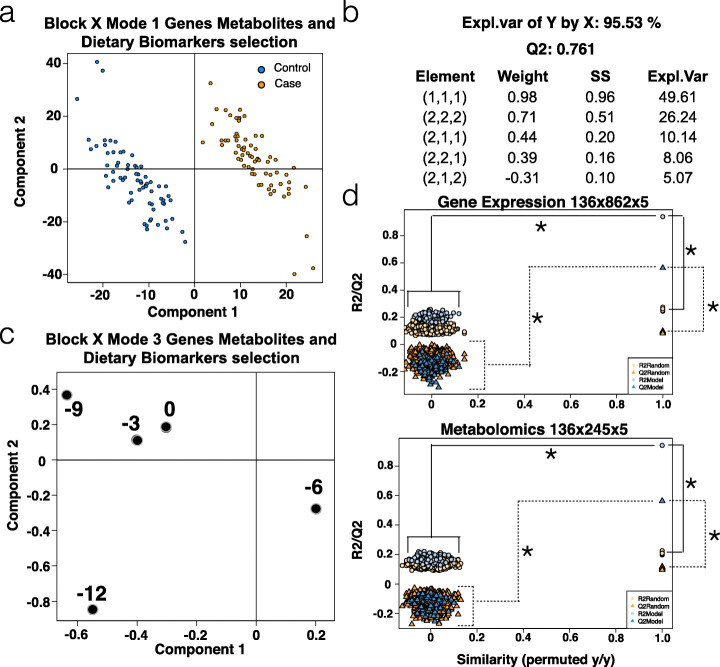
NPLS-DA analysis of TEDDY data. **a** NPLS-DA model mode 1 projection with combined gene expression, metabolites, and DBs showing separation between cases and controls. **b** *R*^2^, *Q*^2^, and element values for the NPLS-DA model. Each element is a triad containing the combination of mode components that captures the indicated explained variance. **c** NPLS-DA model mode 3 projection showing the relevance of early time points in the model as they have high absolute values. **d** VIP variable selection-validation by permutation analysis. For both gene expression and metabolomics datasets, the selected VIP-based features have significantly higher *R*^2^ (circles) and *Q*^2^ (triangles) values compared to a randomly selected set of features, (*) *p* < 0.001

The NPLS-DAs calculated for transcriptomics, metabolomics, and dietary biomarkers (DB) data for the TEDDY cohort resulted in models that explained between 40 and 94% of the autoimmunity phenotype. Some of these models were able to successfully distinguish islet autoimmunity (IA) cases from controls (Additional file 13: Figs. S2-S6). Feature selection by variable importance for projection (VIP) resulted in the identification of 862 genes, 245 metabolites (91 from mass spectrometry, 91 negative-ion lipids, and 63 positive-ion lipids), and 3 dietary biomarkers (vitamin C, vitamin D, and tocopherol) that differed in the time leading up to seroconversion (complete feature list provided as Additional file 12). An integrated NPLS-DA model, which included all the selected features (Fig. 2), was able to distinguish the outcome in 95.53% of subjects with a predictive capability LOOCV *Q*^2^ of 0.761 (Fig. 2a). Fivefold and 10-fold cross-validation strategies yield the same performance results (Additional file 13: Fig. S7). The model retained most of the explained variance in the first (1,1,1) and second (2,2,2) elements (Fig. 2b). The third mode revealed high absolute values 12 and 9 months before seroconversion (MBSC), indicating that these time points retained most of the disease-related information (Fig. 2c). Assessment of variable selection significance revealed that, in both scenarios, the model with VIP-selected variables produced significantly higher *R*^2^ and *Q*^2^ values (Fig. 2d). Evaluation on a set of independent samples not used for variable selection showed that the predictive capability of the PLS-DA models reached 88% at 12, and 66% at 9 MBSC (Additional file 13: Fig. S8a), and the classification success was over 0.8 when 12-month samples were used to predict outcome at 12 and 9 MBSC (Additional file 13: Fig. S8b).

### Enrichment and partial correlation analysis of the IA progression signature

**Fig. 3:**
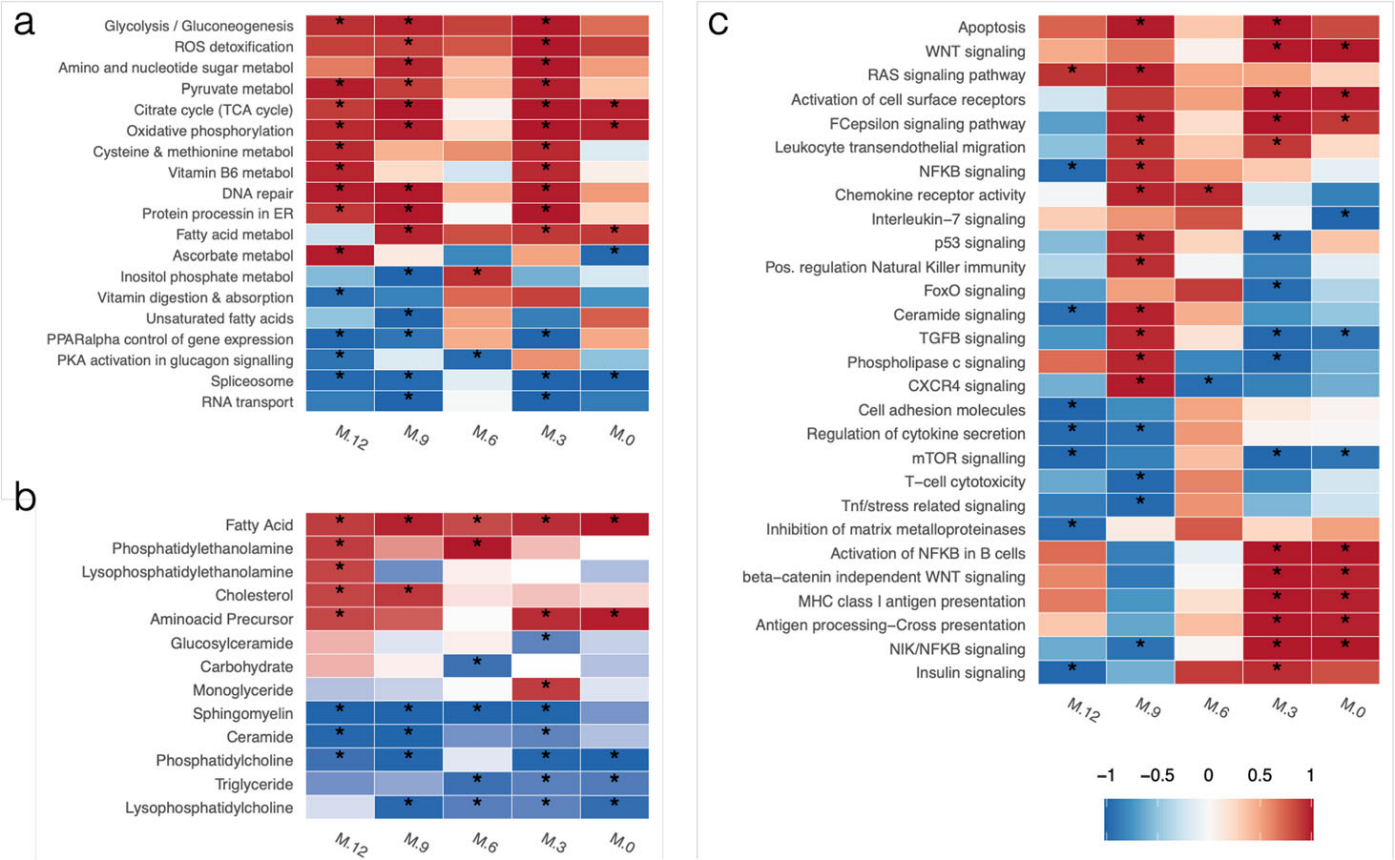
Heatmap of enriched pathways and metabolite groups before seroconversion. **a** KEGG metabolic pathways. **b** Metabolite classes. **c** KEGG signaling pathways. Colors indicate mean up- (red) or down- (blue) regulation of the pathway, while color intensity indicates significance level. For each time point, Gene Set Enrichment Analysis of time-specific NPLS-DA VIP loadings was performed, *p* values for each pathway were combined using Fisher’s method and adjusted for multiple testing. Pathway were selected for having a combined, Fisher adjusted *p* value < 0.05 in at least one combination of time points

To assess the biological significance of the selected features, functional enrichment analyses for KEGG pathways and major metabolite classes were performed (Fig. 3, Additional file 14). We identified a distinctive transcriptional signature, wherein metabolic processes related to glucose utilization and energy (*glycolysis, pyruvate metabolism, oxidative phosphorylation, citrate cycle*) were significantly upregulated across most time points. This high-energy state was accompanied by the enrichment of *ROS detoxification* and *DNA repair* genes, suggesting that an oxidative state resulting in cellular damage is present in IA-developing children. Additionally, pathways associated with lipid regulation, such as *PPARα control of gene expression*, and RNA metabolism (*Spliceosome*, *RNA transport*), were mostly downregulated (Fig. 3a). In parallel, the analysis of enriched metabolic classes indicated that *fatty acids*, *cholesterol*, and *phosphatidylethanolamines* were upregulated in IA cases, while *Sphingomyelins*, *Phosphatidylcholines*, *Triglycerides*, and *Ceramides* were downregulated in cases at nearly all studied time points (Fig. 3b). The upregulated metabolites are consistent with a lipid metabolism impairment landscape in IA-onset individuals, as has been reported previously [15]. Moreover, downregulation of ceramides has been associated with skin disorders like ichthyosis and keratosis occurring in two thirds of children with T1D [16], and low levels of sphingomyelin in pancreatic islets of NOD mice during progression to autoimmune diabetes has also been reported [17].

A more complex regulatory pattern was observed when analyzing signaling pathways that were enriched during the 12 months preceding seroconversion (Fig. 3c). While some processes, such as *apoptosis* and *RAS signaling pathway*, appeared to be generally upregulated, many pathways involved in immune system signaling and communication were activated at 9 MBSC and either maintained activation (i.e., *leukocyte transendothelial migration*, *activation of cell surface receptors*, *chemokine receptor activity*) or became downregulated (*regulation of natural killer immunity*, *CXCR4 signaling*, *TGFβ signaling, FoxO signaling*) near the time of seroconversion. Interestingly, some pathways were constantly downregulated (*mTOR signaling*) or only strongly activated at 0–3 MBSC, such as *antigen presentation* (*MHC Class I and cross-presentation*), *insulin signaling*, and *activation of NFkB in β cells*. This pathway activation-repression pattern suggests a succession of signaling events that involve general cell maintenance, immunity recognition, communication, and response pathways that may contribute to progression towards islet autoimmunity.

**Fig. 4:**
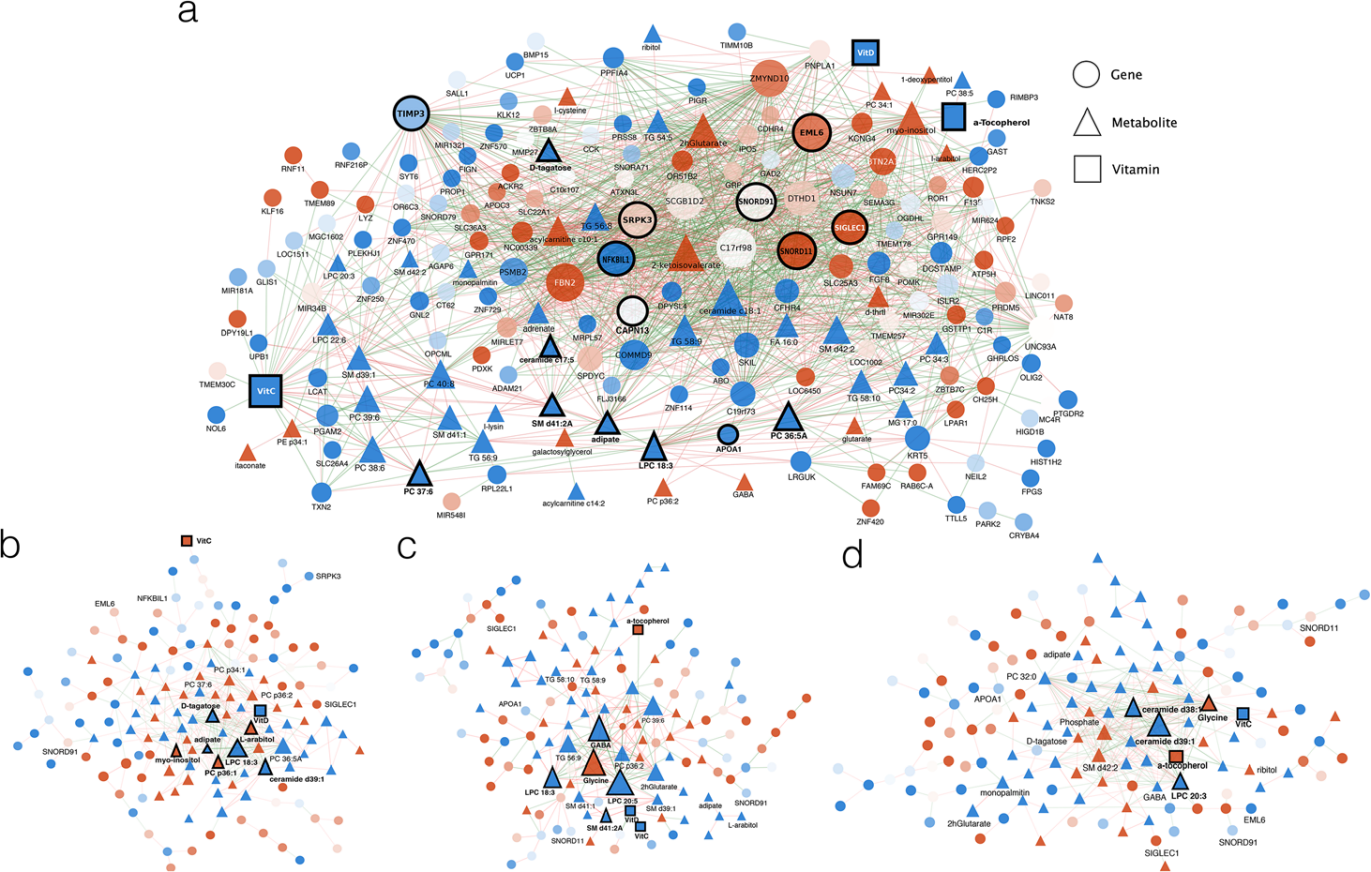
Partial correlation analysis results of the autoimmunity predictive signature evaluated at different timeframes before seroconversion. **a** 12–9 MBSC. **b** 9–6 MBSC. **c** 6–3 MBSC. **d** 3–0 MBSC. Genes, metabolites, and vitamins are represented as circles, triangles, and squares, respectively. Node color indicates mean up- (red) or down- (blue) regulation of the feature in cases at the first network time point. Edges’ color indicates positive (blue) or negative (red) correlation. Only links with absolute partial correlation values > 0.7 are represented

To further investigate the coordination of molecular changes occurring during autoimmunity progression, partial correlation analysis (PCoA) networks were generated for the transition between any two consecutive time points (Fig. 4). To capture a manageable set of most variable features across multiple time points, a variable shrinkage strategy was applied (see “Methods”) resulting in a selection of 315 variables to be included in the networks (Fig. 4). At earlier time points, networks were more densely connected and had a higher representation of gene features (Fig. 4a). As time progressed towards seroconversion, connectivity between genes decreased and more metabolites became integrated into the networks (Fig. 4b–d). Network differences were confirmed by statistical analysis of the distribution of correlation values. Kurtosis analysis indicated that correlation values in the 12to9 network had a platykurtic distribution (kurtosis = − 0.12), indicating many extreme correlation values, while other time points had leptokurtic distributions (0.24, 1.18, and 1.17 for 9to6, 6to3, and 3to0 respectively), indicating fewer high correlation values. Moreover, Kolmogorov-Smirnov tests indicated significant differences between 12to9 and 9to6 (*p* value < 2.2e−16) and between 9to6 and 6to3 (*p value* = 0.03076) networks, while differences between 6to3 and 3to0 networks were not significant (*p* value = 0.1743). These results suggest that a coordinated transcriptional response preceded the metabolic perturbations. These patterns are consistent with our NPLS-DA results, in which the earliest time block (9–12 MBSC) provided information that best predicted the development of autoimmunity. We further analyzed the molecular interaction network at the transition between 12 and 9 MBSC, the period with the highest predictive value in our NPLS-DA model (Fig. 4a and Additional file 13: Table S1). At 9–12 MBSC, network hubs included genes that are characteristic of relevant disease processes. We found genes involved in alternative splicing (*SNORD11*, *SNORD91*, *SRPK3*), a process that we previously showed to significantly impact T1D-related genes [18]. Other hubs included genes involved in macrophage-to-cell adhesion (*EML6*, *SIGLEC1*), regulation of extracellular metalloproteases activity (*TIMP3*), and innate immune responses (*NFkBIL1*). Highly interconnected compounds included vitamin C and D, components of vesicle membranes (APOA1, SM d41:2, LPC 18:3), and intermediate metabolites (Adipate). This result agrees with the functional classes observed in the enrichment analysis and suggests a strong interconnection among energy synthesis, lipid metabolism, nutrient levels, cell signaling, and immune responses in patients that eventually will progress towards autoimmunity.

Together, enrichment and partial correlation analyses reveal a coordinated metabolic and gene expression response that involves sustained changes in energy and lipid components and that is connected to multiple signaling mechanisms leading to immune responses.

### Analysis of the NPLS-DA model for IA progression

While the above enrichment and network analyses revealed major molecular events associated with an autoimmunity predictive signature, these results fall short of enabling a mechanistic interpretation of the pathways to disease. We addressed this challenge by projecting our predictive signature over the template of KEGG pathways using the PaintOmics3 tool, which allows a joint display of gene expression and metabolomics data. When necessary, pathways were edited and combined to include novel associations identified by our partial correlation analysis and to improve readability. The hypothetical model resulting from this interpretive analysis is discussed in the following sections.

### Lipid metabolism irregularities are linked to decreased nutrient absorption, upregulation of glycolysis, and TCA cycle activation in TEDDY IA subjects

**Fig. 5:**
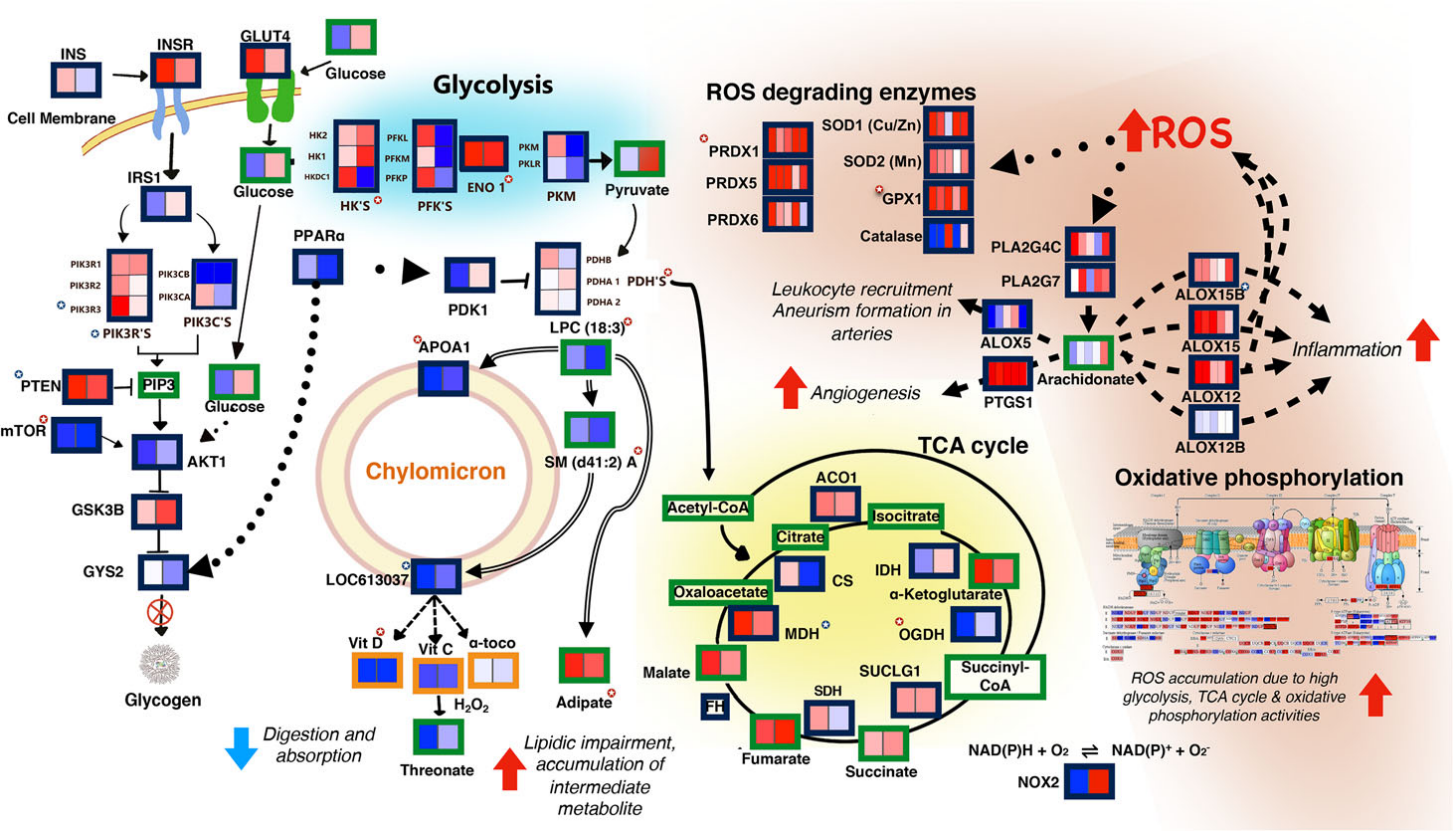
Model of metabolic processes leading to accumulation of reactive oxygen species (ROS) and inflammation in TEDDY IA patients. Low expression of chylomicron membrane components is correlated with secondary metabolites (adipate) and markers of impaired β-oxidation and is consistent with lower vitamin levels and lower expression of the transcriptional regulator *PPARα.* Low *PPARα* levels may downregulate glycogen synthesis and upregulate glycolysis. Increased glycolytic flux is liked to activation of the TCA cycle, increased oxidative phosphorylation, and finally, to the production of ROS. ROS accumulation leads to increased expression of ROS-scavenging enzymes and activation of prostaglandins (PLA2) and lipoxygenases (ALOX) leading to inflammation. Continuous lines: association in KEGG; dashed lines: literature-based association; dotted lines: transcriptional regulation; double lines: partial correlations analysis-based association; blunt ends: negative relationship; arrowheads: positive relationship; red-background stars: VIP-selected variable; blue-background stars: GSEA selected variable; green borders: metabolites; blue borders: genes; orange borders: dietary biomarkers. Heatmap description: from left to right from time points 12 to 9 (2 boxes) or from 12 to 0 (5 boxes) months before seroconversion; blue: downregulation, red: upregulation in cases

Intestine-to-blood lipid and vitamin transport are facilitated by lipoprotein particles known as chylomicrons [19]. A key chylomicron transmembrane protein, apolipoprotein A1 (APOA1), was identified in our analysis as a central model element (Fig. 5). At 9–12 MBSC, downregulation of APOA1 correlated with decreases in lysophosphatidylcholine LPC (18:3), sphingomyelin SM(d41:2) A, and the chylomicron transmembrane protein LOC613037. The primary structural lipid constituents of chylomicrons—sphingomyelins, phospholipids, and triglycerides—were decreased in abundance at all time points in case versus control groups (Fig. 3b, Fig. 4). Moreover, the downregulation of *APOA1* and decrease of LPC (18:3) in cases was correlated with an increase in adipate (Fig. 4a), a known marker of impaired β-oxidation of fatty acids [20]. Therefore, our data suggest that the downregulation of structural lipids and chylomicron components is highly correlated with increased adipate levels in patients progressing towards autoimmunity. Furthermore, the low abundance of chylomicron structural lipids is associated with hydrolyzation of high-density lipoprotein (HDL) phospholipids during inflammation, resulting in accumulation of deleterious, oxidized fatty acids and lipid abnormalities [15]. This result may explain the link between chylomicron deficiency and adipate levels and is consistent with the high levels of fatty acids and cholesterol detected in IA subjects (Fig. 3b).

Vitamins C and D and α-tocopherol, nutrients transported by chylomicrons, are part of our predictive signature, with lower levels identified in cases in comparison to controls at 9–12 MBSC (Fig. 4a). Interestingly, downregulation of chylomicron constitutive elements was associated with absorption deficiency of vitamin C and α-tocopherol in NOD mice [21]. Notably, levels of threonate (Fig. 5), the major breakdown product of vitamin C, were similar between cases and controls, suggesting that low vitamin C is not caused by higher degradation rate, but rather, by restricted absorption. Similarly, low levels of chylomicron components were related to vitamin D deficiency in humans [22, 23], and low vitamin D absorption has been linked to lipid metabolism impairment [22, 24–26]. Based on the collective evidence, we concluded that the lower vitamin levels observed in TEDDY IA cases might be associated with a chylomicron dysfunction leading to LMI.

### Transcriptional dysregulation through low PPARα links energy imbalance, lipid impairment, and ROS activation

Another key component of our model is the peroxisome proliferator-activated receptor-*α* (*PPARα*), which displayed lower expression in cases (Fig. 5)*.* This ubiquitous transcription factor regulates fatty acid and glucose metabolism, as well as peroxisomal and mitochondrial fatty acid β-oxidation [27]. The *regulation of lipids by PPARα* pathway was down at 12, 9, and 3 MBSC (Fig. 3c). PPARα is the primary transcriptional regulator of APOA1, which may explain its low levels in cases. Moreover, PPARα regulates glycogen synthesis by modulating the expression of glycogen synthase 2 (GYS2), an enzyme that is downregulated in cases at 9–12 MBSC. *GYS2* downregulation, together with the high expression of glycogen synthase kinase 3-β (*GSK3B*) and the downregulation of its inactivating enzyme AKT serine/threonine kinase 1 (*AKT1*), suggests that the glycogen synthesis pathway is slightly repressed in IA cases compared to their healthy controls (Fig. 5). PPARα is also a transcriptional activator of pyruvate dehydrogenase kinase isoform 1 (*PDK1*), a major repressor of glycolysis that was downregulated in our data. Notably, metabolic processes related to glucose utilization and energy synthesis (*glycolysis*, *pyruvate metabolism*, *oxidative phosphorylation*, *TCA cycle*) were significantly enriched and activated in cases at most time points before seroconversion (Fig. 3a). IA subjects exhibited elevated expression of several glycolysis-associated genes (Fig. 5), which presumably should result in increased flux through the glycolytic pathway, leading to pyruvate and acetyl-CoA synthesis. Acetyl-CoA, a precursor to both the TCA cycle and fatty acid synthesis, reacts with the TCA cycle intermediate succinyl-CoA to produce adipate, but can also be transformed into more complex fatty acids. Neither acetyl-CoA nor succinyl-CoA was measured in the study; however, the observed accumulation of their anabolic products, adipate, and fatty acids, along with the higher level of TCA metabolites measured, is suggestive of exacerbated TCA cycle in IA subjects. Additionally, upregulation of oxidative phosphorylation (OP) pathway genes was observed in IA subjects at all time points (Fig. 3a). Elevated OP enzyme activities are known to be associated with increased production of ROS. In agreement, we detected enrichment of detoxification process pathways (*ROS detoxification* and *DNA repair*, Fig. 3a) and upregulation of genes encoding intracellular ROS-scavenging enzymes, including glutathione peroxidase (*GPX1*), peroxiredoxin (*PRDX1*), and superoxide dismutase (*SOD*) (Fig. 5).

Collectively, this molecular profile suggests a model in which IA-developing subjects display an imbalance in lipid metabolism that is linked to reduced nutrient uptake, triggers activation of glycolysis, and leads to intermediate metabolites and ROS accumulation as early as 12 MBSC.

### ROS accumulation disrupts arachidonic acid metabolism and leads to an inflammatory response

We detected higher expression levels of several arachidonate-lipoxygenase genes like *ALOX12*, *ALOX15*, *ALOX15B*, and prostaglandin synthase1 (*PTGS1*), known to be activated upon ROS accumulation [28, 29] (Fig. 5). These enzymes transform arachidonate into a variety of proinflammatory and pro-angiogenic molecules such as prostaglandins and eicosanoids [28]. Arachidonate is the result of phospholipases A2 (PLA2s), whose activity is also induced by ROS. The gene expression data indicated upregulation of two phospholipase isoforms, *PLA2G4C* and *PLA2G7*, in cases at multiple time points before seroconversion. Interestingly, we did not detect a significant change in arachidonate between cases and controls, which may indicate a rapid turnover between biosynthesis and catabolism. However, our data did suggest the participation of the arachidonate metabolism pathway in the inflammatory response observed in IA cases. This involvement is also supported by previous works showing that *ALOX12* was implicated in pancreatic inflammation induction and T1D disease progression [30] and that *ALOX5* was involved in leukocyte recruitment and aneurysm formation in arteries [31]. These data reinforce the influence of high ROS levels on the initial inflammatory response as well as chronic inflammation experienced by IA-progressing subjects.

### TNFα activation by ROS accumulation links metabolic imbalance to the observed immune response marks

**Fig. 6:**
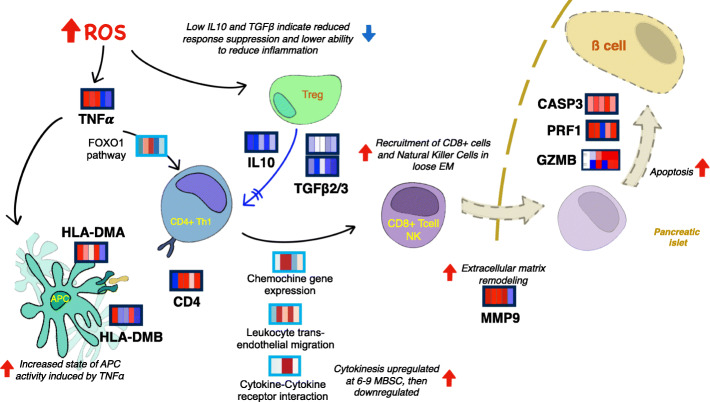
Proposed model of autoimmune processes revealed by IA-predictive multi-omics signature. High ROS levels of IA-developing individuals may induce early upregulation of TNFα, leading to increased activity of APC and the activation of Th cells through FOXO1 pathway. Low levels of TGFβ and IL10 may indicate inability to control T cell proliferation, with high MMP9 suggesting an environment favorable to cell migration. This may result into inflammation, cytokinesis, and migration of immune (CD8+ T or NK) cells to the pancreatic islet, which may provoke apoptosis activation through PRF1/GZMB/Casp3 pathway. Refer to Fig. 5 for more meanings of used symbols. Heatmaps represent either gene expression levels (dark blue boxes) or pathway enrichment data (light blue boxes). From left to right, 5 time points (12, 9, 6, 3, and 0 months before seroconversion) are represented. Blue indicates downregulation and red indicates upregulation in cases

Further interpretation of our multi-omics predictive signature suggests possible links between the observed metabolic dysregulation, ROS-related inflammatory events, and T1D-related autoimmune responses. The data indicated early upregulation of tumor necrosis factor-α (TNFα) (Fig. 6), known to be an effector of T1D development and to be synthesized in response to elevated ROS levels [32]. TNFα activates forkhead box protein O1 (FOXO1) through the PI3K-AKT1/3-FOXO1 pathway, which was found upregulated at 6–9 MBSC (Fig. 3c). In mice, FOXO1 is required for inhibition of T cell activity and its deficiency has been associated with spontaneous T cell activation and differentiation into T-helper1 (Th1) and Th2 cells [33]. Thus, the low *FOXO1* expression at 9–12 and 0–3 MBSC may indicate spontaneous activation and maturation of Th cells in cases. We also observed upregulation of *HLA-DMA/B* genes at 12 and 3 MBSC, and significant activation of antigen presentation pathways at 3–0 MBSC (Fig. 3c). HLA-DM is a heterodimer (1α and 1β chains) and is an invariant MHC protein in humans involved in loading peptides onto MHC class II molecules [34]. Notably, PI3K-AKT1/3-FOXO1 pathway also stimulates the synthesis of HLA-DM isoforms [34]. The changes in HLA-DM levels within antigen-presenting cells (APC), in turn, have been shown to influence the presentation of autoantigens and the development of autoimmune disorders such as T1D [35], which is additionally supported by the pathway enrichment data showing a late upregulation of *antigen presentation* via *MHC Class I* and *Antigen processing−Cross presentation* (Fig. 3c). Therefore, the transcriptional signature found in the TEDDY IA-progressing individuals may represent a process by which ROS-mediated TNFα activation can be linked to Th activation and higher APC activity operating in the development of autoimmunity (Fig. 6).

Additionally, significant downregulation of the transforming growth factor-beta (*TGFβ*) and interleukin-10 (*IL10*) genes was observed (Fig. 6). TGFβ and IL10 are produced in monocytes and T-regulatory cells (Tregs) and serve to regulate the proliferation of T-helper cells. This regulation is critical for the maintenance of self-tolerance and immune homeostasis as well as for suppression of global immune response and inflammation [36, 37]. Thus, low expression levels *TGFβ* and *IL10* in cases suggest a failure to sufficiently suppress immune response and inflammation.

### IA-developing patients show changes in extracellular matrix components and increased immune-cell mobility

The enrichment analysis revealed pathways implicated in chemotaxis (*chemokine gene expression*, *cytokine-cytokine receptor interaction*) and immune-cell migration (*leukocyte transendothelial migration*) (Fig. 3c) to be upregulated in cases at 3–9 MBSC. This indicates the activation of immune system communication processes months ahead of the actual detection of islet autoantibodies. Chemotaxis could also be favored by increased levels of phosphatidylethanolamines at 6–12 MBSC, which are known to play a role in the disassembly of contractile rings during cytokinesis [38]. Moreover, our predictive signature included the upregulation of metalloproteinase 9 (MMP9) at 3–12 MBSC, which could facilitate cell migration and autoimmune response by extracellular matrix remodeling. In agreement, proteomics studies have identified both innate immunity and extracellular matrix proteins as T1D biomarkers [8, 39].

Simultaneous with the upregulation of *MMP9*, the metalloproteinase inhibitor *TIMP3* was downregulated at early time points (Fig. 6). *TIMP3* was one of the most interconnected genes (Fig. 4, Additional file 13: Table S1), correlated at 12–9 MBSC with *SIGLEC1*, an immunoglobulin superfamily member that facilitates macrophage-to-cell and macrophage-to-lymphocyte adhesion [40], and *NFKBIL1*, a divergent member of the IκB family that is involved in the negative regulation of innate immune response [41] (Fig. 4). Altogether, these biomarker expression signatures suggest that cases may display a more permeable extracellular matrix (ECM) that would facilitate the cytokinesis-dependent cell migration processes previously described (Fig. 6).

### Processes disrupting 훽 cells might be detectable on peripheral blood mononuclear cells (PBMC) samples of IA subjects

Since our blood multi-omic signature revealed T cell activation and immune-cell migration processes in IA-developing children, we searched for markers of β cell destruction in our data. Blood biomarkers, such as the islet autoantibodies, may be indicative of distal organismal pathologies, which liberate molecular signals into the bloodstream. The upregulation of apoptosis-associated genes, including perforin (PRF1), granzyme B (GZMB), and caspase3 (Casp3), was identified in cases throughout the 12 MBSC (Fig. 6). The PRF1/GZMB/Casp3 pathway is associated with CD8+ T- and Natural Killer-mediated destruction of antigen-presenting β cells [42]. Notably, a significant enrichment of the Natural Killer-mediated destruction pathway at 6 MBSC was also detected by our analyses (Fig. 3c). These transcriptional profiles may reflect a progression towards IA that involves a pro-apoptotic autoimmune process initiating β cell degradation at early time points. This may be followed by increased activity of Natural Killer and/or CD8+ T cells at 6 MBSC, and finally, antigen presentation via MHC1 and MHC2 at 0–3 MBSC leading to the appearance of autoantibodies as markers of IA seroconversion in the blood (Fig. 6).

Altogether, our analysis of immune processes revealed by the blood multi-omics signature associated with IA progression lends support to a model in which ROS stimulates increased activity of antigen-presenting cells and decreased activity of Tregs within an environment that favors immune-cell patrol, activation of autoimmunity, and migration of cytotoxic-CD8+ T and NK cells towards to pancreatic islets, where they coordinate the degradation of β cells.

## Discussion

High prevalence diseases such as type 1 diabetes are the consequence of the interaction of a variety of genetic and environmental factors that contribute to the establishment of a complex molecular phenotype leading to the onset of the disease. The molecular dysregulation pattern of T1D has been extensively studied via high-throughput assays such as genomics, transcriptomics, metabolomics, and metagenomics. However, few studies measure these different features on the same set of individuals and over an extended period as the TEDDY project does. Such an experimental design allows for an integrative analysis that can reveal the contributions and interactions of different molecular disease factors and their progression to autoimmunity. The realization of such analysis potential requires advanced preprocessing and statistical tools capable of harmonization, modeling, and interpretation of highly heterogeneous data. Here, we addressed these challenges by deploying an analysis pipeline that (a) makes use of the TEDDY case-control design and a time-rescaling strategy to combine measurements from individuals of different age, sex, and genetic background into a comparable and sufficiently powered dataset; (b) models omics features through 3D tensor structures, both separately and jointly, to recover the contribution of each molecular layer to disease progression while providing a unique predictive model; (c) combines multiple bioinformatics pathway analysis methods to propose interpretable models of disease. This analysis strategy strongly contrasts with most multi-omics disease studies where either omics modalities are poorly integrated [43], have no temporal dimension [44], or are restricted to evaluate predictive performance without biological interpretation [45]. We believe the integrative/predictive/interpretative strategy presented here is a valuable contribution to disease modeling that can be applied to other multifactorial pathologies.

We used a multivariate approach (NPLS-DA) and combinatorial variable selection strategy based on the VIP statistics to identify a predictive multi-omics signature. The NPLS approach was preferred for this study as it naturally accommodates the three-dimensional structure of our dataset to return information about patients, features, and the dynamics of the disease progression. Moreover, the VIP-based variable selection results in a predictive signature that can be further analyzed and interpreted by enrichment methods. We note that PLS may be prone to overfitting when the number of variables largely exceeds the number of observations. Possible model overfitting was addressed here both at variable selection and prediction performance, in the first case by implementing a permutation test for the final selected variables (Fig. 2d), and in the second by providing performance values on a set of samples fully excluded from the model build. These calculations indicated that the multi-omics predictive feature was highly significant (0.77) with especially high predictive capacity (0.88) at 12 months before seroconversion (Fig. 2d and Additional file 13: Fig. 7). This 12-month predictive result was surprising, yet highly significant, as it reveals that an altered and disease-distinctive molecular phenotype is already established in IA-progressing children as early as 1 year before the actual detection of beta cell autoantibodies in blood. This finding suggests that disease progression is a process extended over time but, more importantly, it opens a window of opportunity for therapeutic intervention.

We present an interpretative analysis of the NPLS-DA autoimmunity predictive signature that combines time-resolved enrichment analyses to identify disease-evolving cellular processes, partial correlation analysis to unravel novel molecular associations, and PaintOmics-assisted data representation to incorporate existing pathway maps into one integrated biological model. The results of the interpretative effort also revealed many interesting patterns. First, we detect a metabolic phenotype in IA-developing children that is sustained during the analyzed 12-month period before seroconversion. This phenotype consists of the upregulation of energy-producing pathways and fatty acids, and the downregulation of structural lipids, triglycerides, and a major transcriptional regulator of lipid metabolism. Different elements of the metabolic signature reported in this work have been previously described in the literature as having links with T1D. For example, the metabolic stress before seroconversion has been reported to impact levels of phospholipids and energy metabolites [10, 46]. Low levels of lysophosphatidylcholine and sphingomyelins were related to T1D [17], but also to HDL phospholipid levels during inflammation, accumulation of oxidized fatty acids, and disruption of the structure of APOA1 [47]. Similarly, low levels of APOA1 have been associated with lipid abnormalities in young T1D subjects [15]. Finally, vitamin D deficiency is a reported marker of islet autoimmunity [22, 23]. Our analysis reports similar behaviors of these features in the same group of individuals, thereby corroborating previous findings. Additionally, our study provides deeper and time-informed mechanistic insights of disease progression where additional features expanding multiple molecular layers are revealed. Combined, we propose a model for TEDDY autoimmune children that stresses the lipid metabolism impairment (LMI) phenotype present at 12 months before seroconversion, which is associated with lower vitamin levels, possibly due to defects in nutrient uptake. Compared to healthy individuals, cases progressing to IA showed lower glycogenesis and higher glycolytic activity, indicating that consumption, rather than storage of glucose, prevails. Products of glycolysis feed into the TCA cycle, which in turn provides substrate for the electron transport chain and oxidative phosphorylation, both processes were strongly upregulated throughout the year before autoantibody detection. High-energy and oxidative state may result in an excess of ATP synthesis, which could explain the observed accumulation of fatty acids, cholesterol, and toxic secondary metabolites like adipate [20]. Besides, upregulated oxidative phosphorylation in cases could lead to the accumulation of reactive oxygen species (Fig. 3), possibly resulting in systemic damage and inflammation [48], as corroborated by the measured high expression of genes coding for ROS-degrading enzymes and phospholipases.

This metabolic signature prevails for months before the detection of autoantibodies while signaling and immune-related mechanisms seem to follow a more complex regulatory pattern in which some pathways are activated at 6–9 months before seroconversion while others become active closer to diagnosis. Here, evidence of ROS-related inflammation, angiogenesis, and immune responses was observed (Figs. 3 and 4). Usually, inflammatory responses occur only in consequence of existing cellular dysfunctions. As both LMI and high ROS levels result in cellular damage, the immune system is likely to respond. High ROS is also known to induce TNFα/FOXO1-related pathways, leading to maturation and proliferation of cytotoxic lymphocytes and effector Th1 cells, which stimulate the synthesis and release of pro-inflammatory cytokines [28]. Our model then proposes that increased activities of APCs and Th1 cells, along with low activity of Tregs, propitiate cytokinesis. An increased ECM permeability allows for enhanced motility and communication between macrophages and other innate immune cells. Overall, this may facilitate the activity of an already in-alert-state immune system, thereby increasing the likelihood of an autoimmune response, and leading recruitment of CD8+ and/or NK cells to the pancreatic islets. Ultimately, this series of events may result in the activation of apoptosis, and consequent destruction of β cells, which is characteristic of T1D (Fig. 4). The activated APC phenotype 3–0 MBSC may accelerate β cell destruction and the ultimate detection of islet autoantibodies, which is the endpoint of our study.

In summary, our analysis proposes a series of events that start as early as 12 months before seroconversion and involve metabolic, inflammatory, and autoimmune processes, inferred by the combination of transcriptomic, metabolomic, and dietary biomarker profiles. While data were obtained from different biological compartments (PBMC and plasma) and their communication has not been modeled, there is overlap in the metabolic processes that are present and active in both. Interestingly, results of recent work indicate a similarity in the overall metabolic profiles of PBMC and plasma of diabetic patients, with differences between cases and controls being larger in PBMC extracts [49]. These results indicate that integrative analysis of PBMC transcriptomic and plasma metabolites is meaningful to elucidate the metabolic and regulatory trends that operate in the bloodstream of affected individuals. Conversely, a benefit of the multi-compartmental nature of this study is that it offers a broad range of insights on the underlying factors of T1D progression at a systems level, as demonstrated by our detection of significant correlations between molecular features of diverse nature.

Additionally, results shown here combine most of the TEDDY cohort in one unique analysis, regardless of important disease factors such as type of first-appearing autoantibody, ethnicity, or country of origin. Unfortunately, further stratification of the multi-omic data based on these factors results in limitations at the effective sample size. Therefore, we acknowledge that the results shown here do not represent a complete disease model nor capture possible differences in disease subtypes. Still, this study demonstrates the power of the integrative approach to model complex disease processes with temporal resolution and to identify molecular disease phenotypes months before the current diagnosis capacity. Ultimately, the results provide information that will guide the development of strategies for early diagnosis and treatment of T1D.

Finally, while our study establishes a metabolically impaired and high inflammatory state in children with HLA risk genotypes who progress towards autoimmunity, it does not provide any insights on the origin of this physiological condition. Causes could be either genetic or environmental. The genetics of T1D has been extensively studied with nearly 50 SNPs found to have a significant association with T1D [50]. T1D genetic risk scores have also been proposed [51]. While many of the identified SNPs are related to genes of the immune system [50], the vast majority of them are non-coding and their regulatory effects remain uncertain. The results of our analyses provide new hypotheses for targeted studies evaluating the extent to which T1D-associated genetic variations impact the metabolic processes described in this study. Similarly, a wealth of literature exist that associate external environmental factors with T1D [52]. For example, viral infections [53], nutritional factors [54–56], and stressful life events [57, 58] have been linked to increased IA and T1D incidence.

However, some other environmental factors showed contradictory results. For example, cow milk intake in childhood has been associated with both an increased risk of IA [59] and T1D [60], and a decreased risk of T1D [61]. More research is definitely needed to delineate the contribution of environmental factors and their interactions with predisposing and/or protective genes to the development of IA and T1D.

The TEDDY study, which also collects lifestyle and exposure data, represents a unique opportunity to analyze the relationship between environmental factors and the molecular phenotypes discovered here. Additional studies have the potential to further elucidate the type and timing of environmental triggers affecting the onset of islet autoimmunity as well as the subsequent course of disease in those who ultimately develop T1D.

## Methods

### TEDDY study design

TEDDY is an international study that enrolled 8676 newborn infants with a high- or moderate-risk class II HLA genotype between 2004 and 2010 [62]. The individuals used in this study were selected based on their HLA-DR-DQ genotype [63], which indicated high risk of developing T1D. Participants are closely followed for the development of IA or type 1 diabetes, with study visits every 3 months from birth to age 48 months, and every 3 or 6 months thereafter. Patients were followed either until development of T1D (30% or enrolled individuals) or to the age of 15 (control individuals). Participating study centers included Georgia/Florida, Colorado, and Washington in the USA, and Finland, Sweden, and Germany. IA cases were defined by confirmed autoantibody positivity to either insulin (IAA), GAD (GADA), or IA-2 (IA-2A) in two consecutive visits, the first of which defines the case’s event age. At each visit, blood samples were taken to profile gene expression, metabolomics, and dietary biomarkers. The study methods were carried out in accordance with the approved guidelines by local Institutional Review or Ethics Boards [62, 64]. Due to the large number of samples involved, specific procedures were applied to minimize batch effect. Briefly, samples from each case and matched controls were run in the same analytic batch. When this was not feasible due the limited number of samples that the batch can process, samples to be compared with each other (i.e., collected at the same visit) were arranged to be run in the same analytic batch [14]. Data are available upon request from the NIDDK Central Repository at https://www.niddkrepository.org/studies/teddy.

### Gene expression

Blood sampling from enrolled children began at 3 months of age, with subsequent samples taken at 3-month intervals for 48 months, after which they were taken biannually. Total RNA was extracted from 2.5 mL peripheral blood per sample using high-throughput (96-well format) extraction protocol that applies magnetic (MagMax) bead technology at the TEDDY RNA Laboratory, Jinfiniti Biosciences in Augusta, GA. Purified RNA (200 ng) was further used for cRNA amplification and labeling with biotin using Target Amp cDNA synthesis kit (Epicenter catalog no. TAB1R6924). Approximately 750 ng of labeled cRNA was hybridized to the Illumina HumanHT-12 Expression BeadChips per the manufacturer’s instructions. The HumanHT-12 Expression BeadChip provides coverage for more than 47,000 transcripts and known splice variants across the human transcriptome. After hybridization, arrays were washed, stained with Cy3-conjugated streptavidin, and scanned. Gene expression data were generated for 306 individuals. The beadarray and lumi Bioconductor packages were used for preprocessing microarray data, including image analysis, quality control, variance stabilization transformation, normalization, and gene annotation. The Median Background method was used for local background correction. Also, the BeadArray subversion of harshlight (BASH) method was used for bead artifact detection, which takes local spatial information into account when determining outliers. Each probe is replicated a varying number of times on each array; the summarization procedure produces bead summary data in the form of a single signal intensity value for each probe. Illumina’s default outlier function and modified mean and standard deviation were used to obtain the bead summary data. Variance-stabilizing transformation (vst) and robust spline normalization (RSN) methods, which combine features of quantile and loess normalization, were applied to correct for batch effect and obtain between-array data normalization. The pairwise structure of the data permits elimination of biases associated with the characteristics of the pair, such as gender, age, and country of origin, as previously described [14]. Quality control leads to exclusion of arrays with any of the following undesirable attributes: corrupted image files, high gradient effects on probe intensities, high percentage of beads masked by the BASH method, low mean or median number of beads used to create the summary values for each probe on each array after outlier removal, low proportion of detected probes, low percentage of housekeeping genes expressed above the background level of the array, gender discrepancies assessed with massiR package, or poor pairwise array correlations.

### Metabolomics

The Fiehn laboratory at the NIH West Coast Metabolomics Center (University of California, Davis) quantified metabolomics abundance measures (metabolites and lipids) for all cases and controls for each available study visit from birth until the case event time. Primary metabolites and complex lipids were quantified from citrate plasma using GC-TOF MS and CSH-QTOF MS data acquisition, respectively, at the NIH West Coast Metabolomics Center at the University of California, Davis [65]. GC-TOF MS data were acquired as previously described [66], with data processing and compound identification using the BinBase algorithm [67]. LOESS followed by batch ratio normalization (QC samples were used to adjust sample batch median to global study median) was performed across all metabolomics samples to estimate and remove analytical variance and batch effect. For complex lipids, samples were extracted by methyl-tert-butyl ether/methanol/water [65], followed by chromatogram peak detection and alignments using Mass Profiler Professional (Agilent, Santa Clara, CA). Peaks detected in a minimum of 30% of samples were aligned, with missing peaks recovered by backfilling strategies. Lipids were identified using the Fiehn laboratory’s LipidBlast spectral library [68]. Briefly, after LOESS signal correction, quality control samples were used to adjust sample batch median to global study median of all samples to reduce batch effect. Normalization was performed across all the samples to estimate and remove analytical variance. GC-TOF metabolomics, positive-ion lipidomics, and negative-ion lipidomics contained 1556, 514, and 443 features respectively. Metabolomics data were generated for 1556 individuals. Further details on the TEDDY metabolomics data can be found at [69].

### Dietary biomarkers

Plasma from blood drawn into light-protected tubes (BD Vacutainer®CPT™ Cell Preparation Tubes) was used to determine dietary biomarkers at the Genomics and Biomarkers Unit at the National Institute for Health and Welfare, Helsinki, Finland. 25(OH) D concentrations were measured using the ARCHITECT 25-OH Vitamin D chemiluminescent microparticle immunoassay (CMIA) [23]. For ascorbic acid measurements, 50 μL plasma was transferred into cryovials and stabilized by adding 0.2 ml of 5% trichloroacetic acid (TCA) plus 200 mg disodium EDTA with subsequent freezing at − 70 °C. Ascorbic acid concentration was determined by an ion-paired, reversed-phase, high-performance liquid chromatographic method using electrochemical detection, as described [70, 71]. Isoascorbic acid was used as internal standard for the quantitation of ascorbic acid. Fatty acids were analyzed from erythrocytes stabilized with 2-propanol and butylated hydroxytoluene. A gas chromatographic method [72] modified from previously published methods [73, 74] was used. Erythrocyte fatty acid composition was analyzed using an Agilent 6890 gas chromatograph (Hewlett Packard, Palo Alto, CA, USA) with a split injector and hydrogen as the carrier gas on a capillary column Omegawax 320 (length: 30 m, I.D.: 0.32 mm, phase layer: 0.25 μm; Supelco, Bellefonte, PA, USA). The percentage composition of fatty acid methyl esters was normalized to 100%. Plasma retinol, carotenoids and tocopherols were extracted from 50 μl of plasma by liquid-liquid extraction using n-hexane and ethanol [75]. First, tocopherols were determined from the samples using HPLC and fluorescence detection, after which retinol and carotenoids were determined by using HPLC and multiwavelength detection, using a modified version of a previously published method [75]. Cholesterol analysis was performed using an enzymatic cholesterol assay on the Architect ci8200 analyzer (Abbott Laboratories, Abbott Park, IL, USA). Dietary biomarker data were generated for 1621 individuals. Further details on the TEDDY dietary biomarker data can be found at [23].

### Dataset construction and binning

For our study, we selected TEDDY individuals for which gene expression, metabolomics, and dietary biomarker data were available for 12 months before seroconversion, representing a total of 306 subjects. Seroconversion was defined as the second successive visit with detection of islet autoantibodies (GADA, IA2A, or IAA). Hence, cases were those individuals who developed autoimmunity during the study, while controls were defined as those who remained disease-free. Cases and controls were matched by age, ethnicity, and collection center. Timepoint zero in the analyses was defined as the date of IA confirmation for each case. Multi-omics data were then binned at 3-month intervals for each case and matching control, resulting in time point intervals 0, 3, 6, 9, and 12 months before seroconversion. The bins were defined as follows: the 0, 3, 6, 9, and 12 time points as 1–1.5, 1.5–4.5, 4.5–7.5, 7.5–10.5, and 10.5–13.5 months before seroconversion. When present, multiple values from the same subject within each bin were averaged.

### Tridimensional data array and Tucker3-based missing value imputation

Each omics dataset was arranged as a tensor or 3-way structure where each mode accommodated for each of the three dimensions involved in the study. Modes 1 to 3 represent individuals, omics variables, and time, respectively, with dimension values □, □, and □. Thus, an element of the array *X*, *x*_*ijk*_ corresponds to the value for patient □ at an omics feature □ at time point □. This structure facilitates the assessment of data covariation patterns to be studied across the three modes of our datasets simultaneously.

Tucker3 is a method for summarizing the three sets of modes constituting the three-way data set in such a way that the main information in the data can be summarized using a limited number of components for each set of modes [76]. Using the aforementioned data structure, the Tucker3 method was used to impute missing values for each dataset independently. To avoid excess imputation, only individuals with values in at least 3 of the 5 time points were included in the analysis; globally, 28% of the total gene expression data and 16.3% of the metabolomics data was imputed. The imputation strategy entailed an iterative fitting procedure in which the mean of the matrix was assigned to the missing values. Next, a Tucker3 model was fitted to the data, and previously imputed values were replaced with the new fitted model values. This was followed by the calculation of the prediction error for non-imputed values. The fitting was repeated until an error convergence value of 1 × 10^− 7^ is reached. This imputation strategy assured that the imputed values had a minimal influence on the model obtained from the true data (Additional file 13: Fig. S8). The best numbers of components for the Tucker3 models were calculated using the DIFFIT procedure [77].

### Case-control within-normalization

Case and controls were matched by age, gender, race, and clinical center [14]. Each case was paired with one control for the transcriptomics dataset and three controls for the metabolomics and dietary biomarker datasets. To leverage the TEDDY case-control structure, while preserving the case-control discriminative labels, a within-normalization procedure was applied to each case-control set. For each case-control set and at each time point, the mean value was obtained and subtracted from each individual measurement. For instances in which multiple controls were available, the mean control value was obtained first and then used to compute the average with the case value. This internal normalization method ensured that high absolute values were obtained when cases diverge greatly from their controls, whereas near-zero absolute values were obtained when small differences were present within the set. After normalization, principal component analysis was used to confirm no residual batch effects were present in the data.

### N-way partial least squares-discriminant analysis (NPLS-DA)

NPLS-DA was performed to determine the variables that best explained the differences between cases and controls. NPLS-DA models were constructed for individuals with complete or imputed data in all three data modalities. NPLS is an extension of PLS to multiway structures [78, 79]. PLS calculates a set of components that maximize the sample covariance between the response and the linear combination of the predictor variables. The function to be solved is:

1 $$ {\mathrm{ArgMax}}_{u_h^{\prime }\ {u}_h=1,{v}_h^{\prime }\ {v}_h=1}={\operatorname{cov}}^2\left({u}_h^{\prime }X,{v}_h^{\prime }Y\right) $$

where *u*_*h*_ is the *h*th (left) eigenvectors of the singular value decomposition (SVD) of X matrix for any *h* PLS dimension, *v*_*h*_ are the *h*th (right) eigenvectors of the singular value decomposition (SVD) of *Y* matrix for any *h* PLS dimension.

Barker and Rayens [80] demonstrated that coupling PLS with linear discriminant analysis (DA) is a successful statistical approach to deal with *p* >> n data structures and with variables with high multicollinearity, as in transcriptomics datasets [80]. The power of NPLS-DA lies in the fact that similarities between groups (cases and controls) across time points are ignored, whereas the differences are magnified, thereby controlling noise inherent to large datasets [80]. This strategy generates a regression model for each response variable based on the predictor *X* variables [81].

For this study, we combined NPLS with DA to create N-dimensional multivariate models that are able to discriminate between two population classes, such as cases and controls. This was accomplished by the singular value decomposition (SVD) of the tridimensional (3D) data array per mode (individual, variables, and time) of the block X (matrix of predictors variables). In this way, one matrix per mode for a total of three matrices was obtained. Each matrix contained scores or loadings depending on the mode being analyzed. Through the DIFFIT approach [77], the number of components per mode that retained most of the information from the 3D structure was extracted and transformed into a manageable two-dimensional representation. Lastly, discriminant analysis is performed with these three (scores = individuals, loadings1 = variables, and loadings2 = time) datasets, allowing for the behavior of each mode to be graphed after the sample classification. The models with the best number of components were iteratively evaluated by leave-one-out cross-validation, in which a sample is excluded to calculate and adjust the goodness of fit (*R*^2^) and the predictive outcome capability (*Q*^2^) of each model. Also, fivefold and tenfold cross-validation were performed (Additional file 13: Fig. S7), obtaining an average *Q*^2^ value around 0.75, similar to the one reported in Fig. 2 obtained with LOO. This demonstrates that the cross-validation method proposed in our work faithfully describes the performance of the model.

The pseudo-code for variable selection and model validation is:

*Split of the samples into 5 or 10 folds, or take (1- total samples) for LOO cross-validation*

*for i in 1:n: # where n could be 5, 10 or (1- total samples)*

*testingSet <- samples in i*

*trainingSet <- samples not in i*

*fit PLS-DA using trainingSet*

*Selected Vars <- Selection of variables that describe best the model*

*Prediction <- Predict class of the samples in i using the fitted model with Selected Vars*

*Performance Evaluation <- Obtain R*^*2*^ *of the model and* *Q*^*2*^ *based on prediction*

*Repeat this process 1000 times*

*Obtain average R*^*2*^ *and Q*^*2*^ *through all iterations and folds*

### Variable selection

A variable selection strategy was designed based on the Variable Importance for Projection (VIP) statistics that quantifies the influence that each predictor variable has over the response variable in terms of the total sum of squares of the model [82]. We applied the VIP in three different data arrangements to capture feature variability. The VIP2D was calculated after deconvolution of the 3D array to a wide matrix of adjacent time points; hence, one VIP value per component per variable at different time points was calculated. In the VIP3D model 1, a mean VIP per variable at all times was calculated, resulting in one VIP value per variable per component, constraining the time mode. In the VIP3D model 2, we first calculated the VIP per variable per time point per component, and then summed and analyzed the mean of all VIP values for a component. For each VIP strategy, predictor variables with values larger than the 99th percentile for gene expression and 95th percentile for metabolomics data were selected. Intersection and union of these features resulted in new sets of features that were again tested in the NPLS-DA models. Thus, the feature dataset with the highest goodness of fit (*R*^2^) and predictive outcome capability (*Q*^2^) values was then chosen as the best model for the omics dataset.

### Model validation

The performance of the NPLS-DA model was evaluated by leave-one-out cross-validation. Additionally, the model was validated both for the significance of the feature selection and for the prediction on an independent set of samples.

To validate the feature selection, a permutation strategy inspired by previous works [83–85] was implemented to assess both the significance of the selected features for predicting the outcome data and the robustness of the outcome label in capturing disease signatures. For the significance of the model, *R*^2^ and *Q*^2^ of the VIP-selected features were compared to the values obtained using a 100× random selection of models with the same number of features to calculate a permutation *p* value of the VIP selection versus random feature selections. To test the robustness of the method, the outcome label across cases and controls was randomly permuted. NPLS-DA models were computed using either our VIP-selected variable set or random feature selections, and again compared *R*^2^ and *Q*^2^. The permutation test demonstrated the significance of the feature selection related to IA.

Additionally, the NPLS-DA multi-omics feature selection was validated on an independent set of samples not used in the model build. These were samples of individuals with complete multi-omics measurements in less than three time points. The validation set contained 8, 6, 18, 31, and 26 samples for time points − 12, − 9, − 6, − 3 and 0, respectively. As the individuals in this validation set do not have data for 5 time points, the NPLS-DA cannot be directly applied. Instead, we used the set of VIP-selected features to predict outcome by linear discriminant analysis, for each time point separately.

### Partial correlation analysis

Partial correlation analysis was performed among the VIP signature to identify associations between features within the TEDDY case-control dataset.


where *X* and *Y* are the variables which correlation is required, controlled by all other Z variables (Z = {Z_1_,Z_2_, …,Z_n_}.

For partial correlation analysis, only normalized case values were used. To capture relationships associated with disease progression, correlations were computed for differences between omics variable values at two consecutive time points. Hence, profiles of 4 time periods were obtained: 12–9, 9–6, 6–3, and 3–0 months before seroconversion. Moreover, an elastic net regression strategy was applied to the VIP signature to select those features with the highest variability at each time window. Briefly, shrinkage analysis from *α* = 0 (Ridge regression) to *α* = 1 (Lasso regression) was performed using all possible intermediate values to determine the best shrinkage method for every particular time period, with 100 iterations for each *α* values from 0 to 1 by 0.1 increments. Next, for each period, features were selected by creating 1000 regression models using their winning *α* value, and features were collected that appeared at least once across repetitions and time. While partial correlation was computed for the full VIP signature, networks were constructed including only the features selected by the described strategy. The network visualization was made using Cytoscape [86], filtering links for absolute correlation values greater than 0.7. Differences between the distributions of correlation values at each time period were evaluated by kurtosis analysis, which measures the shape of the distribution and by Kolmogorov-Smirnov test (KS-test), a non-parametric test that compares the cumulative distributions of two datasets.

### Gene set enrichment analysis

Gene set enrichment analysis (GSEA) was computed using the PIANO R-package [87], ranking genes by their VIP values at each time point. Gene sets were selected from KEGG, Gene Ontology, Reactome, Biocarta, and HMARKS databases. *P* values were calculated for two available modalities, using either the sum or the mean gene expression value as the gene set statistic, and the most significant *p* value was retained. Time point-specific enrichment *p* values were combined using Fisher’s method [88] implemented in the R function *fisher.method()* with Benjamini-Hochberg multiple testing correction. Combined *p* values were obtained for all possible time point combinations and pathways were selected if its Fisher’s method adjusted *p* value was ≤ 0.05 in at least one time point combination. Semantically redundant pathways were manually discarded. Values used to draw enrichment pathways were calculated as 1 minus the *p* value multiplied by the sign of the average expression value of the significant genes present in the pathway.

### Metabolite enrichment analysis

Metabolites were manually assigned to a metabolic class (Additional file 12). Then, differential abundance between cases and controls were calculated with a *t*-test (*p* value < 0.05), and enrichment of metabolic classes among significant metabolites was calculated with Fisher’s exact test. Enriched classes were selected for an FDR corrected *p* value ≤ 0.2. These values were then used to generate a heatmap of metabolite changes across time.

## Supplementary Information

**Additional file 1.** Dietary biomarkers data processed.

**Additional file 2.** Dietary biomarkers data raw.

**Additional file 3.** GCTOF metabolomics data processed.

**Additional file 4.** GCTOF metabolomics data raw.

**Additional file 5.** Gene expression data processed.

**Additional file 6.** Gene expression data raw.

**Additional file 7.** Negative lipidomics metabolomics data processed.

**Additional file 8.** Negative lipidomics metabolomics data raw.

**Additional file 9.** Positive lipidomics metabolomics data processed.

**Additional file 10.** Positive lipidomics metabolomics data raw.

**Additional file 11.** Cohort data.

**Additional file 12.** Selected features.

**Additional file 13.** Supplementary figures.

**Additional file 14.** Pathway enrichment.

**Additional file 15.** Review history.
