# Supplementary material for: Integrative analyses of TEDDY Omics data reveal lipid metabolism abnormalities, increased intracellular ROS and heightened inflammation prior to autoimmunity for type 1 diabetes
Source: Genome Biol. 2021 Jan 21;22:39. doi: 10.1186/s13059-021-02262-w (PMC7818777; doi:10.1186/s13059-021-02262-w)
Supplement: Supplementary file 13 — Additional file 13. Supplementary figures. [file 13059_2021_2262_MOESM13_ESM.pdf]

Supplementary Materials for

Integrative analyses of TEDDY Omics data reveal lipid metabolism abnormalities, increased intracellular ROS and heightened inflammation prior to autoimmunity for type 1 diabetes

Leandro Balzano-Nogueira[1], Ricardo Ramirez[1], Tatyana Zamkovaya[1], Jordan Dailey[1], Alexandria N. Ardissone[1], Srikar Chamala[2], Joan Serrano-Quílez[3], Teresa Rubio[4], Michael J. Haller[5], Patrick Concannon[2,6], Mark A. Atkinson[5], Desmond A. Schatz[5], Eric W. Triplett[1], Ana Conesa[1,6*]

[1]Microbiology and Cell Science Department, Institute for Food and Agricultural Sciences, University of Florida, Gainesville, USA.

[2]Department of Pathology, Immunology and Laboratory Medicine, University of Florida Diabetes Institute, Gainesville, Florida, USA.

[3]Gene Expression and RNA Metabolism Laboratory, Instituto de Biomedicina de Valencia (CSIC), Jaume Roig, 11, 46010 Valencia, Spain.

[4]Laboratory for Neurobiology, Prince Felipe Research Center, Valencia, Spain.

[5]Department of Pediatrics, University of Florida Diabetes Institute, Gainesville, Florida, USA.

[6]University of Florida Genetics Institute, Gainesville, Florida, USA.

*Corresponding author: Ana Conesa, aconesa@ufl.edu.

This PDF file contains:

Supplementary Figs. 1 to 8

Supplementary Table 1

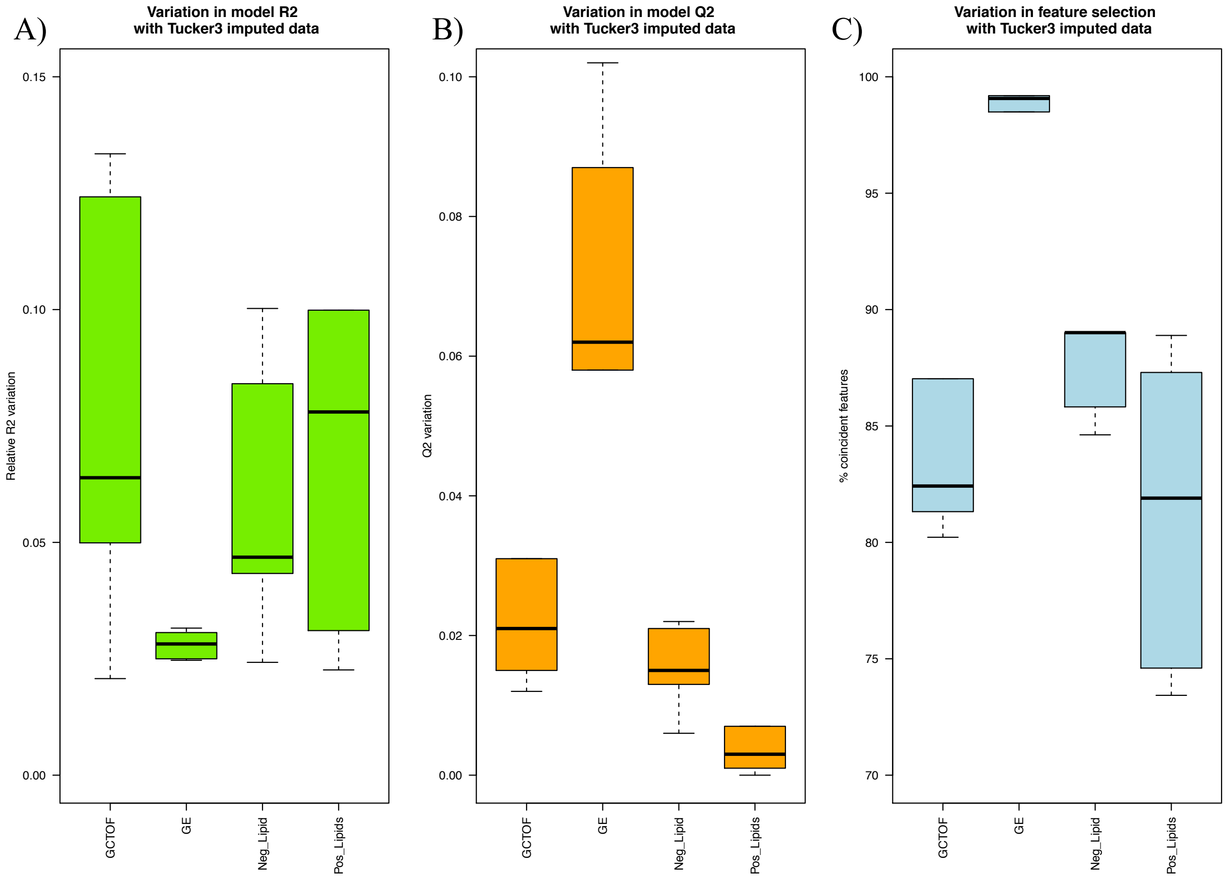

Fig. S1: Evaluation of Tucker3 for missing value imputation. Tucker3 missing value imputation imposes that predicted values have no effect on the multi-way model. From each of the gene expression and metabolomics dataset, five mock datasets were simulated by introducing the number of missing values in the original data at random positions. Tucker3 was used to impute the 3-way tensors, NPLS-DA models were calculated, and variables were selected with the same procedure as original data. Performance (R2) and (Q2) were calculated and the selected feature sets were compared**.** A) Distribution of percentual differences in R2 between our data model and the imputed datasets with random missing values. B) Distribution of differences in Q2 between our data model and the imputed datasets with random missing values. C) Coincidence in variable selection between the true model and the model derived from imputed datasets with random missing values.

A)

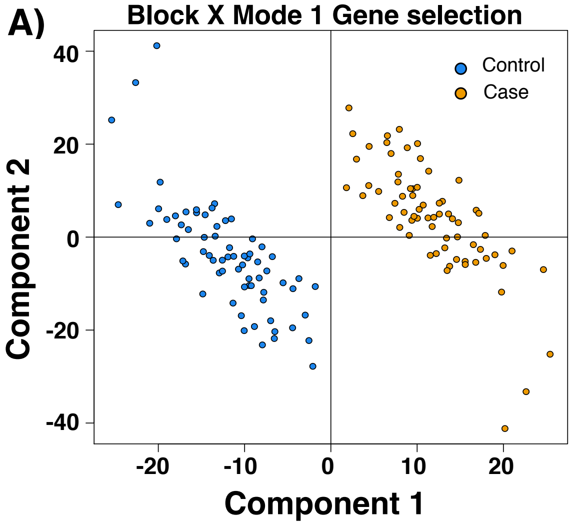

B)

Expl.var of Y by X: 94.02 %

Q2: 0.570

Element	Weight	SS	Expl.Var
(2,2,2)	1.05	1.12	48.79
(1,1,1)	0.96	0.92	39.98
(1,2,1)	-0.32	0.10	4.48
(1,2,2)	-0.28	0.08	3.45
(2,1,1)	-0.18	0.03	1.40

C)

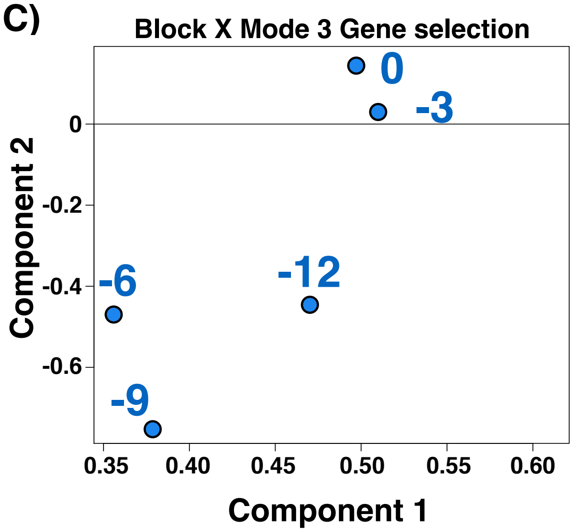

Fig. S2: NPLS-DA analysis of the gene selection data from TEDDY. A) NPLS-DA model mode 1 projection of the 862 genes selected through VIP, showing separation between cases and controls. B) R^2, Q^2 and element values for the NPLS-DA model. Each element is a triad that contains the combination of mode components that captures the indicated explained variance. C) NPLS-DA model mode 3 projection showing the information quantity per time point in the model.

A)

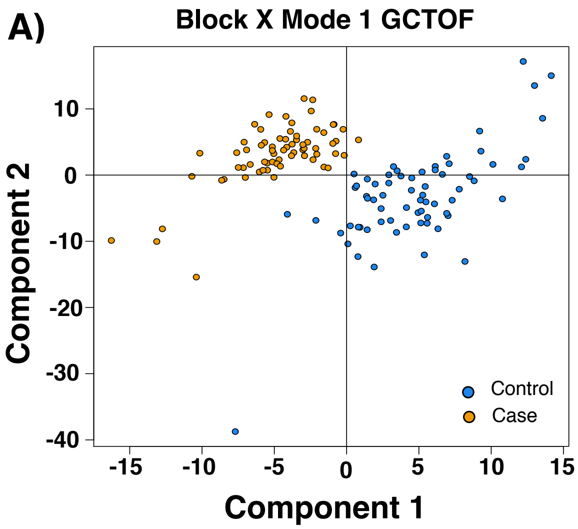

B)

Expl.var of Y by X: 84.37 %

Q2: 0.313

Element	Weight	SS	Expl.Var
(2,2,2)	1.30	1.70	32.33
(1,1,1)	1.26	1.58	30.08
(2,2,1)	-0.81	0.66	12.46
(2,1,1)	0.65	0.43	8.12
(1,1,2)	-0.65	0.42	8.04

C)

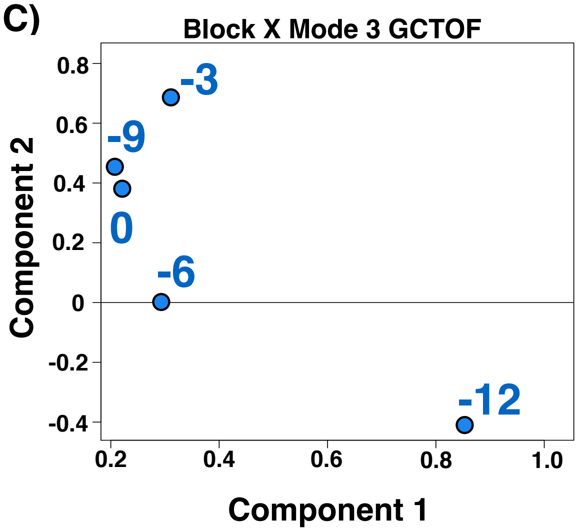

Fig. S3: NPLS-DA analysis of the metabolites selected from GCTOF TEDDY data. A) NPLS-DA model mode 1 projection of the 91 GCTOF metabolites selected through VIP, showing separation between cases and controls. B) R^2, Q^2 and element values for the NPLS-DA model. Each element is a triad that contains the combination of mode components that captures the indicated explained variance. C) NPLS-DA model mode 3 projection showing the information quantity per time point in the model.

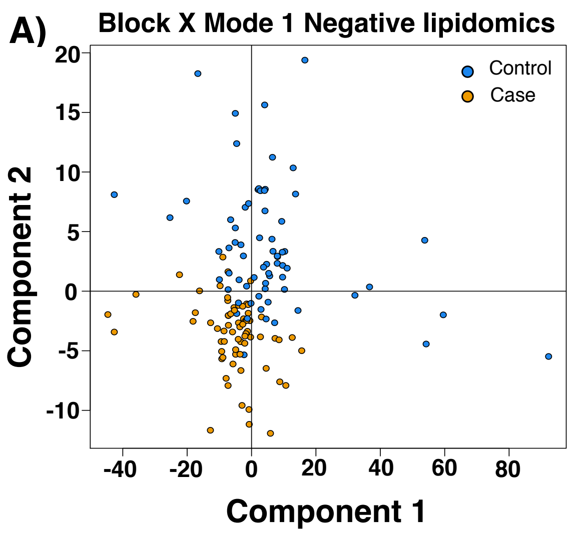

B)

Expl.var of Y by X: 54.47 %

Q2: 0.045

Element	Weight	SS	Expl.Var
(2,1,2)	1.68	2.82	71.16
(1,1,1)	1.00	0.99	25.06
(2,2,2)	0.33	0.11	2.67
(2,1,1)	0.15	0.02	0.54
(1,1,2)	-0.12	0.01	0.37

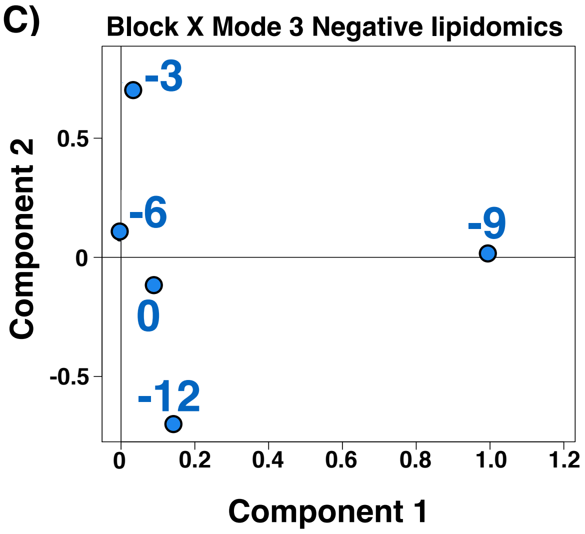

Fig. S4: NPLS-DA analysis of the metabolites selected from negative ion lipids TEDDY data. A) NPLS-DA model mode 1 projection of the 91 negative ion lipids metabolites selected through VIP, showing certain separation between cases and controls. B) R^2, Q^2 and element values for the NPLS-DA model. Each element is a triad that contains the combination of mode components that captures the indicated explained variance. C) NPLS-DA model mode 3 projection showing the information quantity per time point in the model.

A)

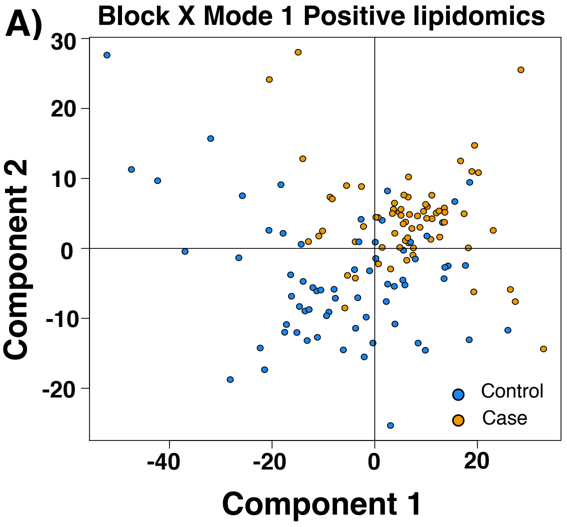

B)

Expl.var of Y by X: 40.25 %

Q2: 0.010

Element	Weight	SS	Expl.Var
(2,2,2)	0.70	0.49	38.13
(1,1,1)	0.55	0.30	23.29
(1,2,1)	-0.50	0.25	19.31
(2,1,2)	-0.34	0.11	8.79
(2,2,1)	0.29	0.08	6.41

C)

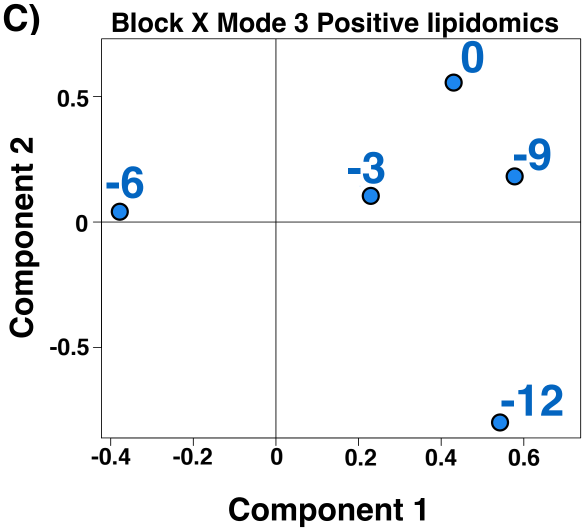

Fig. S5: NPLS-DA analysis of the metabolites selected from positive ion lipids TEDDY data. A) NPLS-DA model mode 1 projection of the 63 positive ion lipids metabolites selected through VIP, showing certain separation between cases and controls. B) R^2, Q^2 and element values for the NPLS-DA model. Each element is a triad that contains the combination of mode components that captures the indicated explained variance. C) NPLS-DA model mode 3 projection showing the information quantity per time point in the model.

A)

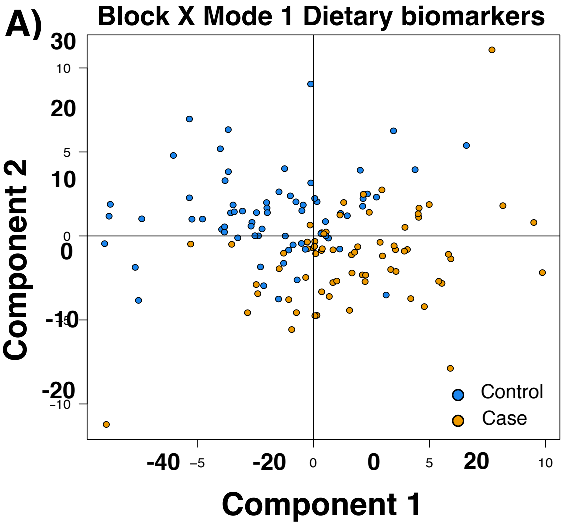

B)

Expl.var of Y by X: 44.73 %

Q2: 0.055

Element	Weight	SS	Expl.Var
(1,1,1)	1.05	1.10	77.51
(1,1,2)	0.36	0.13	8.96
(2,1,1)	0.36	0.13	8.96
(2,2,2)	-0.19	0.04	2.53
(1,2,1)	0.16	0.03	1.85

C)

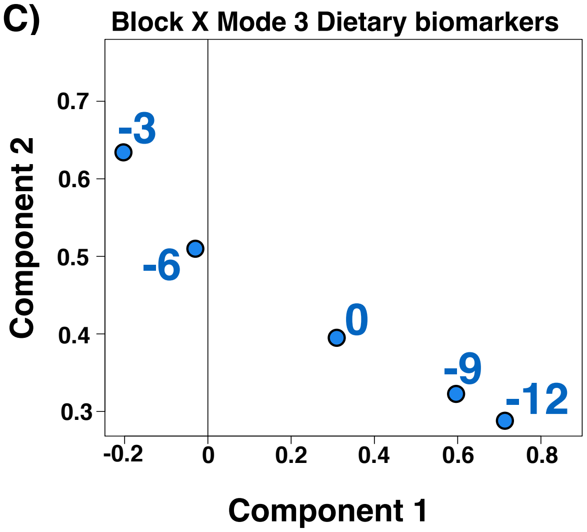

Fig. S6: NPLS-DA analysis of the dietary biomarkers selected from TEDDY data. A) NPLS-DA model mode 1 projection of the 3 dietary biomarkers selected through VIP, showing certain separation between cases and controls. B) R^2, Q^2 and element values for the NPLS-DA model. Each element is a triad that contains the combination of mode components that captures the indicated explained variance. C) NPLS-DA model mode 3 projection showing the information quantity per time point in the model.

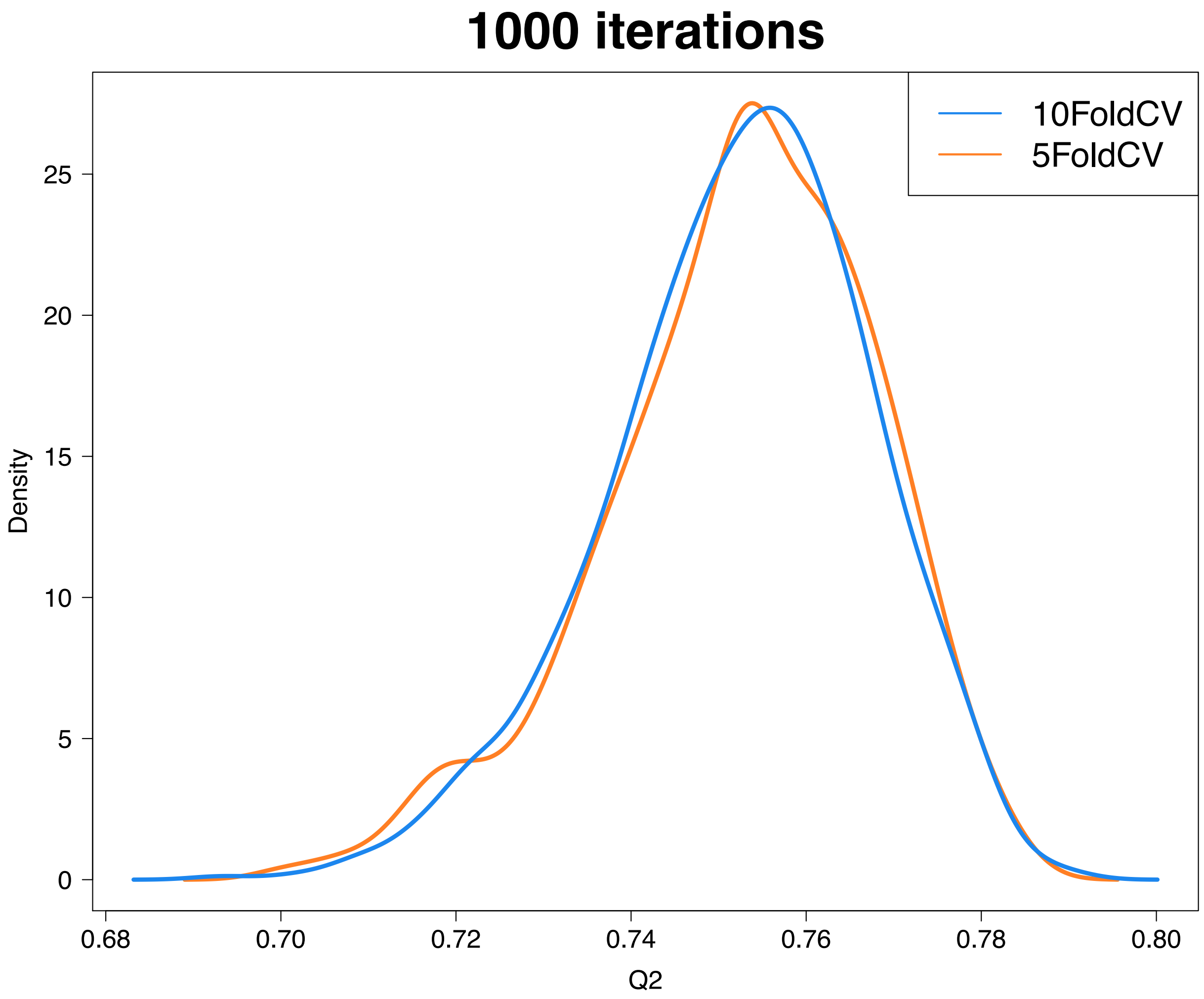

Fig. S7: Model predictive capability evaluated by ten-fold or five-fold cross-validation. Predictive capability (Q2) was calculated as a measure of the performance of the model. One thousand iterations were performed for each (five-fold or ten-fold) cross-validation strategy. The density distribution of the Q2 values demonstrates similar results amongst these two strategies with the original evaluation strategy (leave-one-out) showed in figure 2 of the main article.

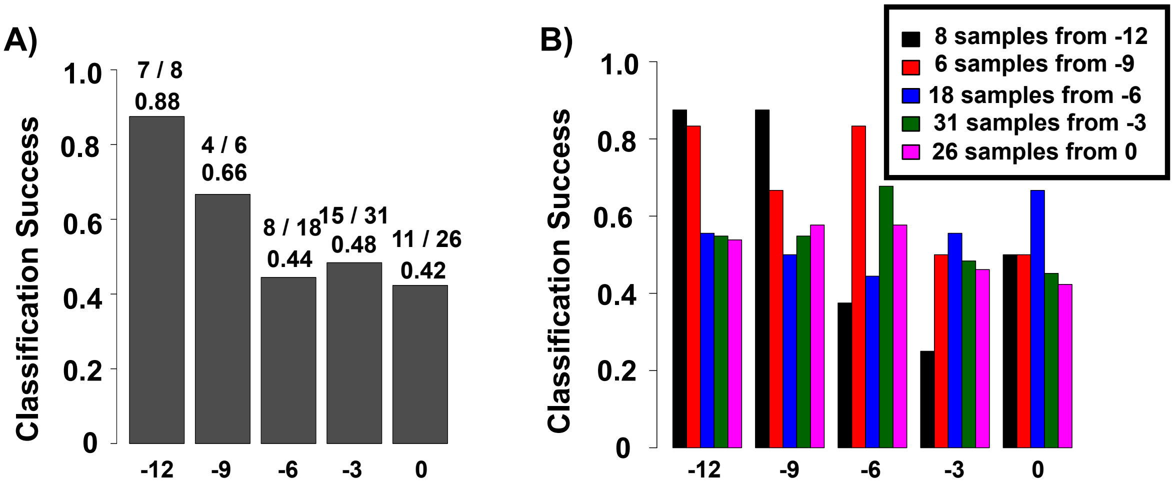

Fig. S8: Evaluation of the predictive capability of the VIP-NPLS-DA selected variables. PLS-DA analyses were performed per time points to predict the samples that were discarded to construct the model due to incomplete time course data (patients with more than 2 missing time points). These samples datasets were predicted using all 1,110 variables selected by the NPLS-DA model. A) Prediction success of each time specific model predicting samples from the same time point. B) Prediction success of the time specific models at samples from each time point.

Table S1: Selected biomarkers revealed by integrative PCoA of multi-omics data from TEDDY IA subjects. To avoid duplicated information, nodes were located in the time frame before SC (in months) in which they converged to a remarkable joint function. (*) Up: upregulated; Down: downregulated; Same: Similar levels, all between cases and controls at the end of the time period.

Time period	Genes (%)	Metabolites (%)	Diet Bioms (%)	|Avg. Corr. Value|	Node Name	Level in cases *	Feature Function	Joint Function
9 to 12	139 (73.5%)	47 (24.9%)	3 (1.6%)	|0.994|	SNORD11<br>SNORD91	Same<br>Up	Involved in RNA splicing (1) and metabolism (2).	Increased rate of alternative splicing (3) and cytokinesis.
					SRPK3	Up	Involved in splicing factors activation related to cell growth (4, 5).	
					TIMP3	Down	Extracellular metalloprotease activity inhibitor (6).	
					EML6	Up	Microtubule elongation contributor (7).	
					SIGLEC1	Up	Immunoglobulin superfamily related to macrophages-cell and macrophages-lymphocytes adhesion. Lupus Biomarker (8).	
					NFKBIL1	Down	Member of the I-kappa-B family of proteins located within the MHC class I region on chromosome 6. It is involved in the negative regulation of innate immune response (9).	
					APOA1	Down	Lipids and vitamins transporter from intestine to blood through chylomicrons (10).	Suggests a synchrony between impaired lipid metabolism, impaired nutrient uptake and accumulation of intermediate metabolites.
					Adipate	Down	Product of fatty acid degradation indicating impaired β-oxidation (11).	
					sphingomyelin SM (d41:2) A	Down	It is associated to several transmembrane and actin-interacting proteins like APOA1.	
					Lysophosphatidylcholine LPC (18:3)	Down	It is positively correlated to sphng57, adipate and APOA1.	
6 to 9	91 (58%)	64 (40.8%)	2 (1.2%)	|0.992|	myo-inositol (my-nstl)	Up	Component of the lipid membrane involved in cell signaling. In diabetes rodent models it is excreted by	

							urine. High levels in blood indicates enhancement of disease progression (12, 13).	
					D-tagatose1 (D-tgts1)	Down	Found in fruits, milk and sweeteners (14). Their consumption may be protective (15, 16).	Markers of decreased insulin sensitivity.
					L-arabitol (l-arbtl)	Up		
					Lysophosphatidylcholine LPC (18:3)	Down	Positive correlation with adipate, phosphatidilcholines (phsph's), ceramides (cermd's), myo-inositol (my-nstl), D-tagatose1 (D-tgts1), l-arabitol (l-arbtl) and benzoic acid (bnzcacd).	
3 to 6	96 (58.5%)	65 (39.6%)	3 (1.9%)	|0.982|	GABA (gmm-mna)	Down	Produced by pancreatic β cells and immune system cells. Although the mechanisms are unknown, high expression levels of GABA lead to partial inhibition of T-cell proliferation in humans (17) and stimulation (18).	These levels promote T-cell proliferation, increasing the disease progression probability.
					Lysophosphatidylcholine LPC (20:5)	Down	Positive correlation with glycine and GABA.	
0 to 3	84 (57.5%)	60 (41.1%)	2 (1.4%)	|0.951|	Ceramide d38:1	Down	Cell recognition (19) and cell signaling (20).	Lower levels of recognition and cell signaling along with a reduced control over blood glucose levels.
					Ceramide d39:1	Down		
					Lysophosphatidylcholine LPC (20:3)	Down		
					Glycine	Up	Stimulator of glucagon release, providing a counterbalancing receptor-based mechanism for controlling α-cell secretory response to glucose levels (21).	

References

1. Kishore S, Khanna A, Zhang Z, Hui J, Balwierz PJ, Stefan M, et al. The snoRNA MBII-52 (SNORD 115) is processed into smaller RNAs and regulates alternative splicing. Hum Mol Genet. 2010;19(7):1153-64.
2. Cavaille J. Box C/D small nucleolar RNA genes and the Prader-Willi syndrome: a complex interplay. Wiley Interdiscip Rev RNA. 2017;8(4).
3. Newman JRB, Conesa A, Mika M, New FN, Onengut-Gumuscu S, Atkinson MA, et al. Disease-specific biases in alternative splicing and tissue-specific dysregulation revealed by multitissue profiling of lymphocyte gene expression in type 1 diabetes. Genome Res. 2017;27(11):1807-15.
4. Wang H-Y, Lin W, Dyck JA, Yeakley JM, Songyang Z, Cantley LC, et al. SRPK2: A Differentially Expressed SR Protein-specific Kinase Involved in Mediating the Interaction and Localization of Pre-mRNA Splicing Factors in Mammalian Cells. The Journal of Cell Biology. 1998;140(4):737–50.
5. Gammons MV, Fedorov O, Ivison D, Du C, Clark T, Hopkins C, et al. Topical antiangiogenic SRPK1 inhibitors reduce choroidal neovascularization in rodent models of exudative AMD. Invest Ophthalmol Vis Sci. 2013;54(9):6052-62.
6. Lei H, Hemminki K, Altieri A, Johansson R, Enquist K, Hallmans G, et al. Promoter polymorphisms in matrix metalloproteinases and their inhibitors: few associations with breast cancer susceptibility and progression. Breast Cancer Res Treat. 2007;103(1):61-9.
7. Li Q, Wojciechowski R, Simpson CL, Hysi PG, Verhoeven VJM, Ikram MK, et al. Genome-wide association study for refractive astigmatism reveals genetic co-determination with spherical equivalent refractive error: the CREAM consortium. Human Genetics. 2015;134:131–46.
8. Biesen R, Demir C, Barkhudarova F, Grun JR, Steinbrich-Zollner M, Backhaus M, et al. Sialic acid-binding Ig-like lectin 1 expression in inflammatory and resident monocytes is a potential biomarker for monitoring disease activity and success of therapy in systemic lupus erythematosus. Arthritis Rheum. 2008;58(4):1136-45.
9. Chiba T, Matsuzaka Y, Warita T, Sugoh T, Miyashita K, Tajima A, et al. NFKBIL1 confers resistance to experimental autoimmune arthritis through the regulation of dendritic cell functions. Scand J Immunol. 2011;73(5):478-85.
10. Imaizumi K, Fainaru M, Havel RJ. Composition of proteins of mesenteric lymph chylomicrons in the rat and alterations produced upon exposure of chylomicrons to blood serum and serum proteins. J Lipid Res. 1978;19(6):712-22.
11. Mortensen PB. Urinary excretion of C4--C10-dicarboxylic acids and antiketogenic properties of adipic acid in ketogenic-stimulated rats due to diabetes, long-chain and short-chain monocarboxylic acids. Biochim Biophys Acta. 1981;664(2):335-48.
12. Kawa JM, Przybylski R, Taylor CG. Urinary chiro-inositol and myo-inositol excretion is elevated in the diabetic db/db mouse and streptozotocin diabetic rat. Exp Biol Med. 2003;228(8):907-14.
13. Baxter MA. The role of myo-inositol in the pathogenesis of diabetic complications. Trends in Endocrinology & Metabolism. 1991;2(5):187-90.
14. Petersen-Skytte U. Tagatose. In: H. Mitchell e, editor. Sweeteners and Sugar Alternatives in Food Technology: Blackwell Publishing, Oxford, UK.; 2006.

15. Kwak JH, Kim MS, Lee JH, Yang YJ, Lee KH, Kim OY, et al. Beneficial effect of tagatose consumption on postprandial hyperglycemia in Koreans: a double-blind crossover designed study. Food Funct. 2013;4(8):1223-8.
16. Donner TW, Magder LS, Zarbalian K. Dietary supplementation with d-tagatose in subjects with type 2 diabetes leads to weight loss and raises high-density lipoprotein cholesterol. Nutr Res. 2010;30(12):801-6.
17. Dionisio L, Jose De Rosa M, Bouzat C, Esandi Mdel C. An intrinsic GABAergic system in human lymphocytes. Neuropharmacology. 2011;60(2-3):513-9.
18. Prud'homme GJ, Glinka Y, Hasilo C, Paraskevas S, Li X, Wang Q. GABA protects human islet cells against the deleterious effects of immunosuppressive drugs and exerts immunoinhibitory effects alone. Transplantation. 2013;96(7):616-23.
19. Dowds CM, Kornell SC, Blumberg RS, Zeissig S. Lipid antigens in immunity. Biol Chem. 2014;395(1):61-81.
20. Kitatani K, Idkowiak-Baldys J, Hannun YA. The sphingolipid salvage pathway in ceramide metabolism and signaling. Cell Signal. 2008;20(6):1010-8.
21. Li C, Liu C, Nissim I, Chen J, Chen P, Doliba N, et al. Regulation of glucagon secretion in normal and diabetic human islets by gamma-hydroxybutyrate and glycine. J Biol Chem. 2013;288(6):3938-51.
