# Supplementary material for: Integrative analyses of TEDDY Omics data reveal lipid metabolism abnormalities, increased intracellular ROS and heightened inflammation prior to autoimmunity for type 1 diabetes
Source: Genome Biol. 2021 Jan 21;22:39. doi: 10.1186/s13059-021-02262-w (PMC7818777; doi:10.1186/s13059-021-02262-w)
Supplement: Supplementary file 15 — Additional file 15. Review history. [file 13059_2021_2262_MOESM15_ESM.docx]

Review History

**First round of review**

**Reviewer 1**

**Are you able to assess all statistics in the manuscript, including the appropriateness of statistical tests used?**

Yes, and I have assessed the statistics in my report.

**Comments to author:**

Balzano-Nogueira et al present a multi-omics analysis of T1D onset. This is a fascinating longitudinal data set that promises to reveal very intersting biology. The data analysis strategy consists of classification (including variable selection), functional enrichment analysis, network analysis and the integration of results into a hypothetical model. In the current form, however the data analysis is not always described in sufficient detail and analysis choices are not always justified. Therefore, it is not yet clear whether all the conclusions drawn are indeed supported by the data.

Major comments:

1) Classification:

An assessment of the classification model is difficult, since the cross validation strategy is not clearly described. In particular, it is not clear, whether the feature selection was included within the cross validation or not. This is crucial, since a feature selection that uses the full data set might lead to overly optimistic performance estimates. Also, a leave one out cross validation might over estimate the actual performance. A clear split in training and test data set (potentially x fold CV) should be done for the evaluating the complete model fit (including classification model and dimensionality reduction / variable selection).

2) Pathway enrichment:

It seems that P-values are not adjusted for multiple testing. Therefore it is currently not possible to judge the reliability of the conclusions drawn from the enrichment analyses.

3) Network analyses:

The network analysis is very descritpive and the interpretation of the network such as analysis of hubs is not supported by statistical analyses: e.g. is alternative splicing enriched among hubs? The conclusions drawn from this analysis are rather generic. Moreover there are multiple steps in the analysis that are not well described or justified (see below).

4) Model:

The hypothetical model that is developed in Figure 5 and 6 is based on the results of the enrichment analysis. Since the validity of the enrichment results has not been established, also the reliability of this model cannot be judged. Also indivual key genes such as APOA1 are described as downregulated, but no statistical significance of this finding is reported.

Minor comments:

5) In the partial correlation analysis it is not clear:

a) why was another feature slection beyind the already slected features necessary?

b) what was regressed in the penalized model?

c) what is the difference between the 1000 models for feature selection?

d) how were partial correlations computed?

e) why were partial correlations computed for the full VIP feature set and then subselected? This makes a difference in the set of variables that partial correlations are condioned upon

f) why was the threshold of absolute partial correlation > 0.7 chosen?

6) The main text does not mention which biomaterial the omics data were derived from. In the methods I gathered, that it is blood. Is blood the relevant tissue for the phenotype? Especially for gene expression? For metboloomics I could not find which tissue it was from.

7) Gene expression data was normalized and corrected for batch effects. How was batch correction performed? What kind of batch structure was there in the data? The authors need to demonstrate that there was no confounding of batch and case control status.

8) Metabolomics data was also processed in batches. The same concerns as for gene expression do apply here. Please demonstrate that no confounding was present and that batch effect removal was effective.

9) Please quantify the missingness in the data, especially in the metabolomics, what was the percentage of samples per metabolite or the percentage of metabolites per sample that were missing?

10) Tucker3: has this method been demonstrated to work for omics imputation? The multi omics factor analysis (MOFA) method seems to adress a very similar problem and has been demonstrated to work in the multi omics setting. The authors should compare their methods.

11) Why was the NPLS-DA approach chosen for classification? (refs 66-69) It seems there are many general purpose machine learning models (including interpretable sparse regression models) available that could be used? The authors should compare their method to other models.

12) From Fig 2 a it seems that cases and controls can be separated perfectly from each other by a linear classifier. Why it the accuracy not 100% then? In leave one out CV this should still be possible?

13) Figure 5: parts of the figure are not readible. Also there should be a legend for the heatmap colors.

**Reviewer 2**

**Are you able to assess all statistics in the manuscript, including the appropriateness of statistical tests used?**

Yes, and I have assessed the statistics in my report.

**Comments to author:**

Balzano-Nogueira et al. used the prospective TEDDy cohort that are HLA-DR-DQ genotype positive. The longitudinal study design is unprecedented.

The authors used a multi-omics approach to understand disease progression over time and identified a signature that is predictive of pancreas islet autoimmunity one year before T1D could be diagnosed.

The authors identified abnormalities in lipid metabolism, a decreased capacity for nutrient adsorption, and accumulation of intracellular reactive oxygen species in children with high genetic risk. The authors also claim that other cellular processes are playing a major role in progressing TID and implicated extracellular matrix remodeling, inflammation, cytotoxicity, angiogenesis, and increased activity of antigen-presenting cells to contribute to

Pancreatic beta-cell destruction.

This is an excellent study making use of the outstanding TEDDY cohort with multiple longitudinal time point and multi-omics investigations with major clinical implications.

I two major point as critique:

1)	As with all multi-omics analyses it would be very helpful if the authors could provide independent validation of their findings. The authors are encouraged to provide these data in independent cohorts and if not possible at least discuss this limitation.

2)	Any level of mechanistic insights & validations are missing. Even though the authors touch this in the discussion it should be highlighted what would be needed to gain mechanistic insights for the hypothesis generated in the TEDDY cohort.

**Authors Response**

**Point-by-point responses to the reviewers’ comments:**

Reviewer #1: Balzano-Nogueira et al present a multi-omics analysis of T1D onset. This is a fascinating longitudinal data set that promises to reveal very intersting biology. The data analysis strategy consists of classification (including variable selection), functional enrichment analysis, network analysis and the integration of results into a hypothetical model. In the current form, however the data analysis is not always described in sufficient detail and analysis choices are not always justified. Therefore, it is not yet clear whether all the conclusions drawn are indeed supported by the data.

Major comments:

1) Classification:

An assessment of the classification model is difficult, since the cross validation strategy is not clearly described. In particular, it is not clear, whether the feature selection was included within the cross validation or not. This is crucial, since a feature selection that uses the full data set might lead to overly optimistic performance estimates. Also, a leave one out cross validation might overestimate the actual performance. A clear split in training and test data set (potentially x fold CV) should be done for the evaluating the complete model fit (including classification model and dimensionality reduction / variable selection).

*Thank you for this remark. We completely agree with the reviewer about the importance of adequate validation and in fact, we have included many different validation aspects in our analysis.*

*1.	The feature selection is indeed part of the validation strategy and therefore variables were selected at each iteration of the cross-validation to obtain the performance of the models.*

*2.	The final variable selection was, additionally, validated in two ways, described in Figure 2 d panels. Here a validation of the selected variables was performed using a permutation strategy, where the R2 and Q2 of selected variables were compared with a random selection of variables. This permutation strategy has been applied in other similar studies (Culeddu, et al., 2012; Wang, et al., 2016; Ahn, et al., 2015) as validation for the feature selection. We have now made reference in our manuscript to this previous work (Page 35, line 22).*

*3.	Once the final model was established, we additionally evaluated the performance of each time-associated set of selected variables on those samples that were left out of the initial model due to incompleteness of the time-course data (as a reminder, we only included in the analysis individuals with measurements at at least 3 out of 5 time points). Although for these samples, we cannot apply the complete time-course defined model, we showed that the time-specific variable selection has a highly predictive value at early time points of the disease progression (-12 and -9 months), which is in agreement with the observed scores of time points in the NPLS model shown in Figure 1c.*

*4.	Finally, leave-one-out cross-validation (LOOCV) was applied as this is a robust validation strategy when, as in our case, a larger k-fold validation will possibly result in a low number of samples being used to learn the model. In fact, LOOCV is recommended when the number of samples is relatively small and when estimated model performance is critical (https://machinelearningmastery.com/loocv-for-evaluating-machine-learning-algorithms/). LOOCV is not recommended for very large datasets or computational expensive algorithms, which is not the case.*

*Therefore, we think that LOOCV is a sound approach to evaluate model performance in our case*

*Having said so, we have taken into account the reviewer's comment and performed 10-fold and 5-fold CV, and calculated the model performance Q2 for a total of 100 iterations in each case. The following figure shows an average Q2 around 0.75 for both 10-fold and 5-fold CV, which is similar to the value obtained with LOOCV. Therefore, we believe that the cross-validation proposed in our work faithfully describes the performance of the model.*

1000 iterations

*·	Culeddu, N., Chessa, M., Porcu, M. C., Fresu, P., Tonolo, G., Virgilio, G., & Migaleddu, V. (2012). NMR-based metabolomic study of type 1 diabetes. Metabolomics, 8(6), 1162-1169.*

*·	Wang, H., Liang, S., Wang, M., Gao, J., Sun, C., Wang, J., ... & Sun, C. (2016). Potential serum biomarkers from a metabolomics study of autism. Journal of psychiatry & neuroscience: JPN, 41(1), 27.*

*·	Ahn, J. K., Kim, S., Kim, J., Hwang, J., Kim, K. H., & Cha, H. S. (2015). A comparative metabolomic evaluation of*

*Behcet’s disease with arthritis and seronegative arthritis using synovial fluid. PloS one, 10(8), e0135856.*

*We include now this result as Supplementary Figure 6, page 7, line 1.*

2) Pathway enrichment:

It seems that P-values are not adjusted for multiple testing. Therefore, it is currently not possible to judge the reliability of the conclusions drawn from the enrichment analyses.

*Thank you for this note. While our initial pathway selection was based on p-values, we required pathways to be significant in at least two out of the five time points of the series, as we considered that consistency of the pathway enrichment across multiple time points increases the significance of the signal. We acknowledge this is not an established approach. Therefore, in order to provide a better formulation for this concept we have calculated combined p.values corresponding to the enrichment at multiple time points using the Fisher’s Method (Fisher, 1925). This approach has been previously used to combine multi-omics data by others (Karathanasis et al 2016) and by us (Hernández-de-Diego et al, 2018) and can be used to generally integrate p.values from multiple hypothesis tests. P.values obtained by the Fisher’s Method can be corrected for multiple testing. Therefore, we have now applied a pathway selection based on the adjusted p.value of the Fisher combined p.value. In order to capture different dynamics of the disease progression, combined p.values were calculated for different time combinations and pathways were selected for having an adjusted p.value < 0.05 in at least one combination (see Methods section, page 37, line 17). Using this approach, we retained most of the initially selected pathways, while additional processes were obtained, such as functions related to RNA processing, ER stress and vitamin digestion and absoption, which are in keeping with other results presented in this paper. We have updated accordingly the Methods section and Figure 3 of the paper. We have also slightly modified the pathway analysis section in results (in blue) to include these additional pathways. Additionally, we provide pathway significance data as Supplementary table SD13 (pag 8 and 9).*

*·	Fisher, R.A. (1925). Statistical Methods for Research Workers. Oliver and Boyd (Edinburgh). ISBN 0-05-002170¬2.*

*·	Karathanasis N, Tsamardinos I, Lagani V. 2016. omicsNPC: Applying the Non-Parametric Combination Methodology to the Integrative Analysis of Heterogeneous Omics Data. PLoS One.Nov 3;11(11):e0165545.*

*·	Hernández-de-Diego R, Tarazona S, Martínez-Mira C, Balzano-Nogueira L, Furió-Tarí P, Pappas GJ Jr, Conesa A. 2018. PaintOmics 3: a web resource for the pathway analysis and visualization of multi-omics data. Nucleic Acids Res. Jul 2;46(W1):W503-W509*

3)	Network analyses:

The network analysis is very descriptive and the interpretation of the network such as analysis of hubs is not supported by statistical analyses: e.g. is alternative splicing enriched among hubs?

*We apologize by not having been sufficiently clear about the purpose and statistical background of the network analysis.*

*First, the network analysis was not intended to identify enriched functional classes, but to further investigate possible co-variation patterns among features selected in our NPLS-DA predictive signature, and possibly to reveal new connections that were not to be captured by a pathway analysis approach, such as relationships between genes and metabolites. In other words, feature selection and functional enrichment were addressed through the NPLS-DA models, and the Partial Correlation Analysis was applied to seek for additional relationships among selected features and evaluate their pattern of change across time-points.*

*The creation of the network is indeed based on a statistical formulation, both for the selection of the features to be shown in the network, as for the method (PCoA) used to build the network and identify links between elements. Therefore, we stress the fact that these networks are the result of a rigorous statistical analysis. As for the biological interpretation of the network, in the paper we discussed two different aspects. One aspect is the number of links across different time points, which is descriptive but interesting as it confirms our NPLS-DA observation about most-coordinated time-points. The other is the discussion of some of the most important hubs. Please note that in our revised version of the manuscript Splicing and RNA transport are enriched pathways among the NPLS-DA selected features.*

*The conclusions drawn from this analysis are rather generic. Moreover there are multiple steps in the analysis that are not well described or justified (see below).*

4)	Model:

The hypothetical model that is developed in Figure 5 and 6 is based on the results of the enrichment analysis. Since the validity of the enrichment results has not been established, also the reliability of this model cannot be judged. Also individual key genes such as APOA1 are described as downregulated, but no statistical significance of this finding is reported.

*We have addressed the issue about the statistical validation of the enrichment analysis in the question above and indicated that all selected pathways have combined adjusted p.values under 0.05, therefore are significantly enriched in the time course. The variable selection, that included APOA1, was performed using the NPLS-DA model described in the first place and also there we demonstrated the statistical significance and predictive capacity of the feature selection. Multivariate statistics are typically not providing single feature level p-values, but a VIP or a loading value that evaluates the importance of the variable for the predictive model. Feature analysis based on multivariate statistics has been widely used in the literature for the analysis of omics data, including for example, in the MOFA approach mentioned by the reviewer. Therefore, the discussed variables are supported by a statistically sound analysis of their relationship with the disease progression. In the case of APOA1 we found to have consistently lower expression in cases than in controls, to be significantly correlated to other variables selected by the model and to be functionally related.*

Minor comments:

5)	In the partial correlation analysis it is not clear:

a)	why was another feature selection behind the already selected features necessary?

*As explained in Q3, the purpose of the additional feature selection at the PCoA was to reduce the size of the network to be manageable, to study the correlation of the same set of elements across time-points, seeking for a biological interpretation of the relationship between them. Note that the goal of the PCoA was not to provide an exhaustive evaluation of all pair-wise correlations, but to identify those connections among the most representative features across time points. In contrast, the goal of the NPLS-DA analysis was to identify a feature set that explains and is predictive for the outcome. Therefore, the additional feature selection prior to PCoA is justified, as it serves a different analysis goal.*

b)	what was regressed in the penalized model?

*Ridge, lasso and elastic net are shrinkage regression methods, they perform variable selection and regularization in order to enhance the prediction accuracy and interpretability of the statistical model it produces. In our case, the response variable was the outcome (confirmed autoimmunity). Explanatory variables were the difference in gene/metabolite expression between consecutive time points. In this way, PCoA focusses on the transition between time-points and the synchronization across molecular features.*

c)	what is the difference between the 1000 models for feature selection?

*Ridge, lasso and elastic net are not deterministic methods, and their results depend on the initiatlization of the algorithm. Therefore, two runs of, i.e. LASSO, may result in a slightly different variable selection. In other to obtain a comprehensive variable selection for the PCoA we run the algorithms multiple (1000) times and retained variables that appeared at least once. This resulted in 315 variables being selected. In this way we guarantee that the shrinkage method returned a set of variables that faithfully represented the variability in the NPLS-DA feature selection and at the same time excluded redundant information.*

d)	how were partial correlations computed?

*Thank you for this question. Please, find below the mathematical formulation of the computation of partial correlation.*

*We have included this formula in the Methods section*

*The zeroth-order partial correlation ρXY·Ø is defined to be the regular correlation coefficient ρXY.*

*It works for any {Z0 }*

*Where X and Y are the variables that we want to calculate its correlation controlled by all other (Z) variables Z = {Z1,Z2,...,Zn}*

e)	why were partial correlations computed for the full VIP feature set and then subselected? This makes a difference in the set of variables that partial correlations are conditioned upon

*This is a relevant question. As indicated by the reviewer, partial correlation computes correlation between 2 variables given other variables in the data. We computed on the full VIP feature set and not after PCoA variable selection because did not want to interfere with the correlation among variables in the VIP feature set, but simply identify and explore those that best represented the variability in the complete set.*

f)	why was the threshold of absolute partial correlation > 0.7 chosen?

*This was an arbitrary threshold that resulted in networks of manageable size. As the goal of PCoA was to evaluate co-expression patterns across time-points and identify hubs, rather than to make inference on every single element resulting network, a reasonable correlation threshold was sufficient for our purposes.*

6) The main text does not mention which biomaterial the omics data were derived from. In the methods I gathered, that it is blood. Is blood the relevant tissue for the phenotype? Especially for gene expression? For metboloomics I could not find which tissue it was from.

*We apologize for the lack of clarity here. All transcriptomics, dietary biomarkers and metabolomics data were obtained from blood samples taken from children’s consecutive visits. Gene expression used whole blood while dietary biomarkers and metabolomics was measured in plasma samples. This is indicated in the Methods section. We have now mentioned that blood tissue in the results section (page 6, line 9). Blood is one of the few tissues that can be easily sampled in longitudinal studies, especially in children. Blood has been used in numerous T1D studies as it captures the immunological, physiological and nutritional state of the patients. As a note, the TEDDY project has published over 100 papers on the analysis of TEDDY blood sample data, and over 250 papers are found in Pubmed under the keywords T1D + blood + “gene expression”*

7) Gene expression data was normalized and corrected for batch effects. How was batch correction performed? What kind of batch structure was there in the data? The authors need to demonstrate that there was no confounding of batch and case control status.

*The TEDDY dataset description paper (Lee, et al., 2014), described the experimental procedures taken at study design to minimize batch effects. These apply to all omics measurements. Briefly, samples from each case and matched controls were run in the same analytic batch. When it was not feasible due the limited number of samples that the batch can process, the consortium arranged samples that were to be compared with each other (i.e., collected at the same visit) to be run in the same analytic batch. This information is now reported in the Methods section of the paper (page 27, line 4). Preprocessing of gene expression data included variance-stabilizing transformation (vst) and robust spline normalization (RSN) methods to further correct any residual technical noise. Moreover, in our data analysis approach, case samples and matched controls were nomalized against each other, thereby technical and demographics effects are cancelled out. Still, before proceeding with statistical analysis, we verified that our normalized measurements were free or any batch effect. This was achieved by PCA of the omics normalised values. Figure below shows PCA of gene expression data with samples colored by experimental batch. No bach effect is observed. We have now included mention to this batch effect issue in the Methods section (page 27, line 4 and 33, line 9)*

*References:*

*1.	Lee, H. S., Burkhardt, B. R., McLeod, W., Smith, S., Eberhard, C., Lynch, K., Hadley, D., Rewers, M., Simell, O., She, J. X., Hagopian, B., Lernmark, A., Akolkar, B., Ziegler, A. G., Krischer, J. P., & TEDDY study group (2014). Biomarker discovery study design for type 1 diabetes in The Environmental Determinants of Diabetes in the Young (TEDDY) study. Diabetes/metabolism research and reviews, 30(5), 424–434. https://doi.org/10.1002/dmrr.2510*

*2.	Sun, H., & Wang, S. (2013). Network-based regularization for matched case-control analysis of high-dimensional DNA methylation data. Statistics in medicine, 32(12), 2127-2139.*

*3.	Johnson, R. K., Vanderlinden, L., DeFelice, B. C., Kechris, K., Uusitalo, U., Fiehn, O., ... & Toppari, J. (2019). Metabolite-related dietary patterns and the development of islet autoimmunity. Scientific reports, 9(1), 1-11.*

8) Metabolomics data was also processed in batches. The same concerns as for gene expression do apply here. Please demonstrate that no confounding was present and that batch effect removal was effective.

*We used metabolomics data normalized according to Johnson et al. 2019 Basically, LOESS followed by batch ratio (QC samples were used to adjust sample batch median to global study median) normalization was performed across all the samples to estimate and remove analytical variance. We refere to the original publication for further details on the preprocessing of these data.*

*References:*

*·	Johnson, R. K., Vanderlinden, L., DeFelice, B. C., Kechris, K., Uusitalo, U., Fiehn, O., ... & Toppari, J. (2019). Metabolite-related dietary patterns and the development of islet autoimmunity. Scientific reports, 9(1), 1-11.*

9)	Please quantify the missingness in the data, especially in the metabolomics, what was the percentage of samples per metabolite or the percentage of metabolites per sample that were missing?

*For gene expression data, we had a 28% of missing samples, while for metabolomics we had a 16% of missing samples. For non-missing metabolomics sample, the overall % of missing metabolites was 0.2%, meaning that most missing in metabolomics data were the missing samples. Please note that the level of missingness is reported in the methods section (page 32, line 9).*

10)	Tucker3: has this method been demonstrated to work for omics imputation? The multi omics factor analysis (MOFA) method seems to address a very similar problem and has been demonstrated to work in the multi omics setting. The authors should compare their methods.

*The reviewer raises a valid concern on the utilization of Tucker3 for missing value imputation. Tucker 3 has been shown to work in the analysis of omics data in a paper from 2010 from our group, although not for the imputation of missing values. However, the imputation by Tucker3 is done in a way that the imputed values have no effect on the model prediction (Walzack and Massart, 2001). In order to demonstrate this property, we applied Tucker3 to mock datasets where we introduced the same amount of missing values as in our original dataset. Once missing values where imputed, NPLS-DA models were calculated, and we applied the same variable selection procedure as described in the paper and compared results. This analysis was done for Gene Expression and each of the Metabolomics datasets. Results are shown below.*

*We observe that variations both in R2 and Q2 are small and that the large majority of the feature selection set is maintained, thereby validating the utilization of Tucker3 imputation in our analysis strategy.*

*Unfortunately, we have not been able to run MOFA due to consistent crashing of the software in up to three different computers, including the HiperGator cluster of the University of Florida. However, direct comparison to MOFA might not be meaningful as MOFA is aimed feature-based imputation, while the Tucker3 approach is designed to have minimal effects on the multi-way modelling.*

11)	Why was the NPLS-DA approach chosen for classification? (refs 66-69) It seems there are many general-purpose machine learning models (including interpretable sparse regression models) available that could be used? The authors should compare their method to other models.

*The goal of our study was to understand the course of molecular events leading to IAA autoimmunity. We used NPLS-DA as it provides a model that has both predictive as interpretative value. By being a latent space analysis approach, the model captures co-variance patterns in the data and is able to recover the features that best represent these variation patterns. This gives a variable selection that is sufficiently large to move ahead into interpretation, for example by pathway analysis. Moreover, by being a regression approach, the predictive value of the model (Q2) provides a means for evaluating significance of the interpretative models. Additionally, NPLS-DA is unique for being able to accommodate the 3-dimensional structure of the data where we simultaneously evaluated omics features, patients and time, thereby facilitating the study of the dynamics of the disease progression. Sparse regression models, even if they return a clear variable selection, will seek sparsity, i.e. a minimum set of variables with high predictive value, thereby complicating the pathway interpretation of the model. Moreover, they are not as suited to reveal dynamics patterns as the NPLS modelling is.*

*To show this point, we have applied sparse logistic regression to our data, as suggested by the reviewer, with LASSO variable selection. We used the R functions glmnet() and with alpha = 1 and lambda parameter estimated by cv.glmnet(). For this, we concatenated all five points in one set of variables. Note that this increased by 5 the number of variables in the model, thereby increasing the risk of overfitting. To be comparable with other model, we applied a similar overall strategy as in our approach: first variable selection for each omics modality was run independently using 5-fold cross validation. We estimated the models 100 times and used the variables that appeared in at least 20% of the iterations, as selected variables to build a multi-omics model. The results of this approach are shown in the table below.*

*The first result that stands out is the reduced number of selected variables in each omics modality, and specially, the low number of overlapping variables at multiple iterations. This is the result of both sparsity and overfitting. Secondly, accuracy is not improved, with a final average accuracy of 0.79, which is similar to that obtained from NPLS-DA.*

*As our manuscript is not a methods paper, including an extensive validation to alternative approaches is out of the scope of this paper. We have included in the discussion of the paper a section that discusses why NPLS-DA is a suitable approach for the interpretive analysis of T1D progression, in comparison with other sparse logistic regression approaches (Page 21 lines 5)*

12)	From Fig 2 a it seems that cases and controls can be separated perfectly from each other by a linear classifier. Why it the accuracy not 100% then? In leave one out CV this should still be possible?

*The representation in Figure 2 is the result of the NPLS-DA model on the actual data, and in this case total separation between cases and controls is obtained. Please, note that the R2, which the goodness of fit of the model is 0.95. This is what you can appreciate in Figure 2. However, high R2 does not always imply that the predictive performance on new samples will be the same high. This is evaluated by the Q2 parameter calculated by cross-validation. This predictive capacity is in our case 0.76 for the complete model.*

13)	Figure 5: parts of the figure are not readable. Also there should be a legend for the heatmap *colors. We apologize for the lack of clarity. We have now edited figure 5 to make sure is fully readable*

Reviewer #2:

Balzano-Nogueira et al. used the prospective TEDDy cohort that are HLA-DR-DQ genotype positive. The longitudinal study design is unprecedented.

The authors used a multi-omics approach to understand disease progression over time and identified a signature that is predictive of pancreas islet autoimmunity one year before T1D could be diagnosed.

The authors identified abnormalities in lipid metabolism, a decreased capacity for nutrient adsorption, and accumulation of intracellular reactive oxygen species in children with high genetic risk. The authors also claim that other cellular processes are playing a major role in progressing TID and implicated extracellular matrix remodeling, inflammation, cytotoxicity, angiogenesis, and increased activity of antigen-presenting cells to contribute to Pancreatic beta-cell destruction.

This is an excellent study making use of the outstanding TEDDY cohort with multiple longitudinal time point and multi-omics investigations with major clinical implications.

I two major point as critique:

1)	As with all multi-omics analyses it would be very helpful if the authors could provide independent validation of their findings. The authors are encouraged to provide these data in independent cohorts and if not possible at least discuss this limitation.

*Thank you for this important remark. As acknowledged by the reviewer, the TEDDY is a unique dataset, where multi-omics measurements were consistently taken for an extended period in the life of children at T1D risk. The study has a case-control structure that allows for control of demographic, gender and age effects. Unfortunately, these study design in unique as the it is the generated data. Therefore, the analysis cannot be replicated in an independent cohort. We add now in the discussion further mention to the uniqueness of the TEDDY dataset*

*However, we believe that our results are backed both with internal controls (please look to answer 1) to Reviewer 1) as externally, since many of our findings pertaining pathways, vitamins and lipids have been separately described in previous reports. We refer to these throughout the paper and in the discussion we acknowledge this fact (“Different elements of the metabolic signature reported in this work have been previously described in the literature as having links with T1D”, page 22 line 7) and highlight that the added value of our study is being able to integrate them into one unified picture.*

*Here we list some of the findings in our analysis with previous literature support*

| ***Feature*** | ***Our paper*** | ***Confirming literature*** |
| --- | --- | --- |
| *Fatty acids, Cholesterol, and Phosphatidylethanolamines* | *upregulated* | *consistent with a lipid metabolism impairment (Fekih et al., 2014)* |
| *ceramides* | *downregulation* | *associated with skin disorders like ichthyosis and keratosis occurring in two-thirds of children with T1D (Pavlovic et al., 2007).* |
| *sphingomyelin* | *downregulation* | *increases progression to*  *autoimmune diabetes in NOD mice (Holm, 2018).* |
| *SNORD11, SNORD91, SRPK3,* | *At 9-12 months before seroconversion, network hubs* | *alternative splicing showed to significantly impact T1D-related genes(Newman et al., 2017).* |

| *Adipate* | *increase* | *marker of impaired 13-oxidation of fatty acids (Mortensen, 1981)* |
| --- | --- | --- |
| *chylomicron* | *Low levels* | *ssociated with absorption deficiency of Vitamin C and α-tocopherol in NOD mice (Fahrmann et al., 2015).* |
| *PPARα* | *Regulatory roles in fatty-acid metabolism, glycogen, Glycolisis* | *Sugden, Bulmer, Gibbons, Knight, & Holness, 2002; Mandard et al., 2007; Kersten, 2014* |
| *APOA1* | *Downregulation* | *Low levels of APOA1 have been associated with lipid abnormalities in young T1D subjects (Fekih et al., 2014)* |
| *TNFα* | *Upregulation* | *an effector of T1D development and to be synthesized in response to elevated ROS levels (Padgett, Broniowska, Hansen, Corbett, & Tse, 2013)* |
| *Vitamin D* | *Downregulation* | *marker of islet autoimmunity(Iqbal et al., 2017; Norris et al., 2018).* |
| *HLA-DM* | *Desregulation* | *influence the presentation of autoantigens and the development of autoimmune disorders such as T1D (Lich, Jayne, Zhou, Elliott, & Blum, 2003* |
| *Porfirin (PRF1), granzyme B (GZMB), and casase3 (Casp3)* | *Upregulation* | *associated with CD8+ T- and Natural Killer-mediated destruction of antigen-presenting 13-cells (Lieberman & DiLorenzo, 2003).* |
| *metalloproteinase 9* | *Upregulation* | *extracellular matrix proteins as T1D biomarkers have been identified (Singh et al., 2017; Zhang et al., 2013)* |

2)	Any level of mechanistic insights & validations are missing. Even though the authors touch this in the discussion it should be highlighted what would be needed to gain mechanistic insights for the hypothesis generated in the TEDDY cohort.

*We thank the reviewer for this comment. Please note that our study does include statistical validation of results and provides elements of biological (Figures 3 & 4) and mechanistic (Figures 4 and 6) interpretation of the data.*

*However, we understand that additional discussion as how to gain further mechanistic insights for our hypothesis, would be useful. Our data have demonstrated a sustained inflammatory state with high ROS in insulin autoantibody (IAA) developing children carrying HLA susceptibility alleles. This inflammatory state is associated with a metabolic impairment condition. This makes some clearly testable predictions about what might be observed in tissues from pre-diabetic subjects. However, we do not address the origin of these differences which could be genetic or environmental or arise from the interplay between these two causes. We have also extended the discussion to include consideration of studies needed to investigate the upstream factors leading to this inflammatory phenotype. (page 25, line 8).*

**Second round of review**

**Reviewer 1**

The authors have addressed many of the previous comments. In some cases, however the modifications to the manuscript in response to the comments do not always fully describe, what the authors state in the response. Thefore still some major points still remain unresolved.

Specifically:

1) My previous comment 1): the main concern was to make sure that the feature selection was run within the cross validation to avoid reporting overly optimistic performances. The authors state so in their response, however this does not become clear from the methods nor the results section.

The authors need to make sure that the analysis was along the lines of this pseudo code and describe this accordingly in the text / methods:

split samples into n non overlapping folds

for i in 1:n:

train <- all samples not in fold i

test <- samples in fold i

features <- select features using data from samples in train

prediction <- predict on data from samples in test using the model on the selected features

performance[i] <- evaluate performance metric

Similarly in the leave one out method the n folds is equal to the number of samples.

2) My previous comment 3): My comment was not aimed at the construction of the network, on which the authors commented. I was aiming at the downstream analyses that are performed once the network is constructed.

Two points on the downstream analysis are made by the authors:

- connectivity changes between time points. This statement needs to be supported by statistical analysis. Could it be that it is simply an effect of the arbitrarily chosen threshold of 0.7 leading to FN edges in one of the analyses? There are methods to formally test for differences in correlations and probably also for partial correlations that should be applied to support this statement.

- again the statement on the hub genes should also be supported by statistics: is there enrichment of these functional terms in the hubs?

3) My previous comment 8): The authors did not demonstrate that no confounding was present and that batch effect removal was effective. Please provide a similar figure (e.g. PCA) as you have done for the gene expression data.

**Reviewer 2**

The authors have addressed all of my previous concerns adequately and I have no further comments.

**Authors Response**

**Point-by-point responses to the reviewers’ comments:**

Answers:

1)	My previous comment 1): the main concern was to make sure that the feature selection was run within the cross validation to avoid reporting overly optimistic performances. The authors state so in their response, however this does not become clear from the methods nor the results section.

The authors need to make sure that the analysis was along the lines of this pseudo code and describe this accordingly in the text / methods:

split samples into n non overlapping folds

for i in 1:n:

train <- all samples not in fold i

test <- samples in fold i

features <- select features using data from samples in train

prediction <- predict on data from samples in test using the model on the selected features

performance[i] <- evaluate performance metric

*Thank you for the suggestion. We rephrased the methods and the result section to make this clear. This is the pseudocode of the implemented procedure:*

*Split of the samples into 5 or 10 folds, or take (1- total samples) for LOO cross-validation*

*for i in 1:n: # where n could be 5, 10 or (1- total samples)*

*testingSet<- samples in i*

*trainingSet<- samples not in i*

*fit PLS-DA using trainingSet*

*SelectedVars<-Selection of variables that describe best the model*

*Prediction<- Predict the class of the samples in i using the fitted model with Selected Vars*

*PerformanceEvaluation<- Obtain R2 of the model and Q2 based on prediction*

*Repeat this process 1000 times*

*Obtain average R2 and Q2 through all iterations and folds*

2)	My previous comment 3): My comment was not aimed at the construction of the network, on which the authors commented. I was aiming at the downstream analyses that are performed once

the	network	is	constructed.

Two points on the downstream analysis are made by the authors:

- connectivity changes between time points. This statement needs to be supported by statistical analysis. Could it be that it is simply an effect of the arbitrarily chosen threshold of 0.7 leading to FN edges in one of the analyses? There are methods to formally test for differences in correlations and probably also for partial correlations that should be applied to support this statement.

*Differences in correlation distributions are usually evaluated applying a Fisher transformation to the*

*correlation values and then applying a statistical test for normal data, for example a t-test that would evaluate if the there are differences in the mean values of two correlation distributions. However, partial correlation analysis is an approach that calculates the correlation of two variables controlled by all others, resulting in a distribution of correlation values that is centered around 0 and normally distributed. Therefore, such test to evaluate changes in the distribution is meaningless.*

*In our case, changes in connectivity’s among nodes result in a different shape (rather than mean value) in the distribution of correlation values. This can be evaluated by a kurtosis analysis and by a Kolmogorov-Smirnov test (KS-test).*

*Kurtosis analysis indicates that 12to9 network has a platykurtic distribution (kurtosis = -0.12) while the others have leptokurtic distributions (0.24, 1.18 and 1.17 for 9to6, 6to3 and 3to0 respectively). This indicates that the 12to9 network has a table-like shape (with many extreme correlation values) while the others have a bell-shape.*

*The KS-test compares the cumulative distributions of two datasets. KS-test indicated significant differences between 12to9 and 9to6 (p-value < 2.2e-16) and between 9to6 and 6to3 (p.value = 0.03076) networks, while differences between 6to3 and 3to0 networks were not significant (p-value = 0.1743).*

*We have added these statistical tests to the results and methods sections.*

- again the statement on the hub genes should also be supported by statistics: is there enrichment of these functional terms in the hubs?

*The statement about hubs genes is descriptive and only notes that some hubs include genes that belong to enriched functional categories of the predictive signature. However, we do not claim that there is an enrichment of hub genes in any functional category. Therefore, we do not think there is a need to include any statistical test here.*

3) My previous comment 8): The authors did not demonstrate that no confounding was present and that batch effect removal was effective. Please provide a similar figure (e.g. PCA) as you have done for the gene expression data.

*We apologize for not having included the batch analysis before. As it is stated in the paper, the metabolomics and dietary biomarker data has been already published and we used the preprocessing procedure applied by the data providers. Therefore, preprocessing of the data was already validated by this previous publication. However, we are including in this response the PCA of metabolomics and dietary biomarker data showing batch labelling, as we did for gene expression data.*
